# Using RT-qPCR, Proteomics, and Microscopy to Unravel the Spatio-Temporal Expression and Subcellular Localization of Hordoindolines Across Development in Barley Endosperm

**DOI:** 10.3389/fpls.2018.00775

**Published:** 2018-06-13

**Authors:** Azita Shabrangy, Valentin Roustan, Siegfried Reipert, Marieluise Weidinger, Pierre-Jean Roustan, Eva Stoger, Wolfram Weckwerth, Verena Ibl

**Affiliations:** ^1^Department of Ecogenomics and Systems Biology, University of Vienna, Vienna, Austria; ^2^Core Facility Cell Imaging and Ultrastructure Research, University of Vienna, Vienna, Austria; ^3^Department of Applied Genetics and Cell Biology, University of Natural Resources and Life Sciences, Vienna, Austria; ^4^Vienna Metabolomics Center, University of Vienna, Vienna, Austria

**Keywords:** barley endosperm, hordoindoline, spatio-temporal, laser microdissection, label-free shotgun proteomics, RT-qPCR, reference genes, grain hardness

## Abstract

*Hordeum vulgare* (barley) hordoindolines (HINs), HINa, HINb1, and HINb2, are orthologous proteins of wheat puroindolines (PINs) that are small, basic, cysteine-rich seed-specific proteins and responsible for grain hardness. Grain hardness is, next to its protein content, a major quality trait. In barley, *HINb* is most highly expressed in the mid-stage developed endosperm and is associated with both major endosperm texture and grain hardness. However, data required to understand the spatio-temporal dynamics of *HIN* transcripts and HIN protein regulation during grain filling processes are missing. Using reverse transcription quantitative PCR (RT-qPCR) and proteomics, we analyzed *HIN* transcript and HIN protein abundance from whole seeds (WSs) at four [6 days after pollination (dap), 10, 12, and ≥20 dap] as well as from aleurone, subaleurone, and starchy endosperm at two (12 and ≥20 dap) developmental stages. At the WS level, results from RT-qPCR, proteomics, and western blot showed a continuous increase of *HIN* transcript and HIN protein abundance across these four developmental stages. Miroscopic studies revealed HIN localization mainly at the vacuolar membrane in the aleurone, at protein bodies (PBs) in subaleurone and at the periphery of starch granules in the starchy endosperm. Laser microdissetion (LMD) proteomic analyses identified HINb2 as the most prominent HIN protein in starchy endosperm at ≥20 dap. Additionally, our quantification data revealed a poor correlation between transcript and protein levels of HINs in subaleurone during development. Here, we correlated data achieved by RT-qPCR, proteomics, and microscopy that reveal different expression and localization pattern of HINs in each layer during barley endosperm development. This indicates a contribution of each tissue to the regulation of HINs during grain filling. The effect of the high protein abundance of HINs in the starchy endosperm and their localization at the periphery of starch granules at late development stages at the cereal-based end-product quality is discussed. Understanding the spatio-temporal regulated HINs is essential to improve barley quality traits for high end-product quality, as hard texture of the barley grain is regulated by the ratio between HINb/HINa.

## Introduction

In barley, hordoindolines (HINs) are described as PIN ortholog proteins. Genetic studies have associated HIN proteins to both major endosperm texture and grain hardness (Darlington et al., 2001; Takahashi et al., 2010). The *HIN* gene family comprises the *HINa*, *HINb1*, and *HINb2* genes (Darlington et al., 2001). *HIN* transcripts are endosperm specific; their mRNA level increased from 14 to 20 days after pollination (dap) in the endosperm and in the aleurone, and declined at 30 dap. Both *HINa* and *HINb* transcripts are more abundant in the endosperm than in the aleurone between 14 and 20 dap. However, no evidence was found that HINa plays a role in grain texture in barley as it was described for PINa (Darlington et al., 2001).

The protein family of puroindolines (PINs) has been affiliated with endosperm texture and grain hardness (Giroux and Morris, 1998). PINs are small, basic, cysteine-rich, seed-specific proteins (Blochet et al., 1993; Gautier et al., 1994). Both PINa and PINb are characterized by a tryptophan-rich domain, that is thought to be responsible for the interaction with neutral polar lipids (Blochet et al., 1993; Dubreil, 1997). PINa and PINb precursors are synthesized with a transit peptide and two additional cleavable propeptides at the N- and C-terminal end. Originally, northern blot analyses of *PINa* and *PINb* during seed development showed an increase for both transcripts till 33 dap and a decrease at mature state (Gautier et al., 1994). *PINa* transcript abundance was always higher than the level of *PINb* transcripts during development and correlated with a higher protein content of PINa compared to PINb in wheat (Blochet et al., 1993). No *PIN* transcripts were detected in *Triticum durum*, indicating the specific expression of PINs in the common hexaploidy wheat *Triticum aestivum* (Dubreil, 1997; Dubreil et al., 1998). In *T. aestivum*, PINs are components of “friabilin,” a complex mixture of neutral and basic polypeptides localized at the starch granule surface and are involved in endosperm softness (Greenwell and Schofield, 1986; Sourdille et al., 1996; Giroux and Morris, 1998). Transgenic rice expressing wheat PINa and PINb reduced seed hardness resulting in reduced starch damage and an increased amount of fine flour particles (Krishnamurthy and Giroux, 2001). Whereas PINa strongly binds to wheat phospholipids and glycolipids, PINb tightly binds only negatively charged phospholipids and forms loose lipoprotein complexes with glycolipids. According to this lipid binding, both PINs play a role in the formation and stability of bread dough foams (Dubreil, 1997). Additionally, PINs were reported to be involved in plant defense mechanisms against plant pathogens (Blochet et al., 1993) where *in vitro* assays show antifungal activity for all PINs (Dubreil et al., 1998). Additionally, transgenic rice plants expressing wheat PINa and PINb demonstrated an inhibition of fungal growth of *Magnaporthe grisea* and *Rhizoctonia solani* after infection (Krishnamurthy et al., 2001). Recent results further showed that PINs affect endosperm development and storage protein polymerization via the protein folding machinery and are thus responsible for the protein matrix formation (Lesage et al., 2011, 2012). In this context, the endosperm texture – and hence, PINa and PINb – affects the properties and quality of flour as the variation of kernel texture influences the milling energy, flour extraction rate, flour particle size, starch damage, pearling quality, malt extract yield, and dry-matter digestibility by ruminants (summarized in Pauly et al., 2013a,b). Microscopic analysis of PIN protein localizations was performed in wheat and oat by immunochemical confocal or by electron microscopy using different PIN antibodies (Gautier et al., 1994; Dubreil et al., 1998; Mohammadi et al., 2006). PINa and PINb localized in the protein matrix surrounding the starch granules in the starchy endosperm. Interestingly, PINa was also found in inclusions of aleurone grains in developing and in mature seeds (Gautier et al., 1994; Dubreil et al., 1998). PIN orthologs have been identified in oats and barley, but were absent in sorghum (*Sorghum bicolor*), maize (*Zea mays*), and rice (*Oryza sativa*; Li et al., 2008). In oat, tryptophanins (TRPs) were identified as PIN orthologs (Tanchak et al., 1998). Expression and localization studies showed that the amount of TRP proteins gradually increased in developing oat seeds and that they are localized at the surface of starch grains in the oat endosperm (Mohammadi et al., 2006).

The cereal caryopsis is composed of different cell layers with distinct, spatio-temporally regulated physiological and molecular mechanisms (Olsen, 2001, 2004). The developmental pathway of cereal endosperm consists of three distinct stages: syncytial, mitotic, and differentiation phase (Olsen, 2004). After differentiation, the fully developed endosperm can account for up to 75% of the seed weight and comprises four major cell types: an epidermal layer of aleurone cells surrounding the starchy endosperm cells, a basal layer of transfer cells, and the cells of the embryo-surrounding region (Olsen, 2001, 2004). The starchy endosperm thereby functions as storage site as it accumulates starch and seed storage proteins (SSPs; Olsen, 2004). The aleurone layer supports seed germination by mobilizing starch and SSP reserves in the starchy endosperm by releasing hydrolytic enzymes that help to degrade the stored nutrients in the endosperm (Olsen, 2004). Maize and wheat have one layer of aleurone cells, rice contains one to several layers, and barley has three layers of aleurone cells (Olsen, 2004). Whereas the persistent inner starchy endosperm cells are dead at the time of full maturation, the aleurone cells are viable and regulate the germination process (Young and Gallie, 2000).

Reverse transcription quantitative PCR (RT-qPCR) and mass spectrometry are high throughput, sensitive, and reproducible methods to quantify the relative abundance of mRNA and proteins, respectively. Normalization by RGs for RT-qPCR is critical and is still one of the most important challenges concerning this technique (Huggett et al., 2005; Ling and Salvaterra, 2011). However, the stability of RGs varies across different cell and tissue types, and even across developmental stages within the same cell or tissue type (Kozera and Rapacz, 2013). Thus, precise selection of RGs for a specific tissue and developmental stage is essential. Ten RGs were characterized for studying gene expression analyses in the developing barley caryopsis (spring cultivar Jersey; Ovesna et al., 2012). However, no characterized RGs are available to measure the relative abundance of mRNA transcripts both at the whole seed (WS) level and in different tissues during barley endosperm development in the barley cultivar Golden Promise (GP).

In this study, we used GP, to investigate the spatio-temporal expression alterations of HINs during endosperm development. The old spring two-row barley cultivar GP is characterized by a high frequency of transformants of immature embryos (Hiei et al., 2014) and is amenable to virus-induced (Holzberg et al., 2002) and RNA-induced gene silencing (Lee et al., 2012). Spatio-temporal microscopy studies describe the SSP pathways in the cultivar GP (Ibl and Stoger, 2014; Ibl et al., 2014).

We harvested GP seeds at four and at two development stages for WS and spatio-temporal RT-qPCR, proteomics, and microscopic studies, respectively, to study the spatio-temporal expression alterations and localization of HINs during barley GP endosperm development. In this context, RGs were validated and characterized to specifically normalize *HIN* transcripts in the WS as well as in distinct tissues during endosperm development. We could observe a constant increase of *HIN* transcripts and HIN proteins during grain development. Using laser microdissetion (LMD), we identified HINa and HINb2 to be most abundant in subaleurone and starchy endosperm at ≥20 dap, respectively. In contrast, *HINb* transcript level was high in subaleurone at 12 dap, indicating a low correlation between transcripts and proteins in subaleurone. HINs are mainly localized to protein storage vacuoles (PSVs) in aleurone, at protein bodies (PBs) in subaleurone and at the periphery of starch granules in the starchy endosperm. In the context of the HIN trafficking pathway, the possible involvement of the endosomal sorting complex required for transport (ESCRT) is presented. Finally, we discuss the putative effects of HINs on the end-product quality.

## Materials and Methods

### Bioinformatics Concerning the RGs and HINs

RG candidates were selected according to their performance (stability values, overall expression degrees) previously reported by Ovesna et al. (2012) and Faccioli et al. (2007). We designed new primers for almost all of those ten RGs [(*SAM* (*S’adenosyl-L-methionine*)) *ELF* (*elongation factor 1-alpha*), *GAP* (glyceraldehyde-3-phosphate dehydrogenase), GRP (glycine-rich protein), *HSP70* (Heat shock 70 kD protein), *ARF* (ADP-ribosylation factor), *FBPA* (fructosebisphosphatealdolase), *HSP90* (heat shock protein 90), *UBI* (ubiquitin gene), *ACPIII* (acyl carrier protein III)] using Primer3 v.0.4.0 (Untergasser et al., 2007; Koressaar et al., 2018) from gene sequences available in the EMBL database resulting in small PCR products (**Supplementary Table S1**). Primer sequences were analyzed using BLAST at IPK Gatersleben homepage^1^ searching for full-length cDNA entries. Additionally, the specific PCR sequences and/or full-length cDNA entries from each RG from the Unigene homepage were analyzed by BLAST using the UniProt platform^2^ searching for the corresponding protein.

We used the *HINs* sequence accession numbers and corresponding primers as previously described (Terasawa et al., 2012). The specificity of the *HINa* and *HINb* PCR products was confirmed by sequencing.

### Plant Material

Barley wild-type variety GP was cultivated as described in (Ibl and Stoger, 2014). We chose four ear development stages for harvesting according to Ibl et al. (2014) and Peukert et al. (2014): one timepoint at prestorage phase (including transition phase) and three timepoints within the storage phase (Peukert et al., 2014). These timepoints include the most dramatic changes of the endomembrane system that is involved in SSP trafficking (Ibl et al., 2014). As the caryopses of the barley ear are heterogeneous in their development at certain developmental stages, we ended up in the following seed development stages: 6–8, 10, 12–18, and ≥20 dap (**Supplementary Figure S1**). RT-qPCR as well as proteomic studies were performed on WS harvested at ear development stage 6–8, 10, 12–18, and ≥20 dap as well as on different cell layers isolated by LMD at 12–18 and ≥20 dap. For spatio-temporal analyses, seeds were harvested from ±20% of the middle of the ear. For the proteomics studies, all seeds from the whole ear were taken to increase the total protein amount. Within this manuscript, we used “6 dap,” “10 dap,” “12 dap,” and “≥20 dap” to describe the stages 6–8, 10, 12–18, and ≥20 dap.

### Tissue Preparation for Laser Microdissection

For LMD microscopy, caryopses between ±20% from the middle of the ear were taken. Embryos of caryopses were dissected and sections from the central region of the grain were excised using a razor blade. They were transferred to a silicon mold (Plano, Wetzlar, Germany), cryogel (Plano, Wetzlar, Germany) was added, and samples were frozen in liquid nitrogen and stored at -80°C. The mold including the grain was fixed to a sample plate, and series of 20 μm sections were cut and immediately mounted on SuperFrost^®^ PLUS slides (Thermo Scientific, Karlsruhe, Germany), which were stored in a Falcon tube containing silica gel to absorb moisture. After 30 min, these dry cryo sections were used for LMD experiments.

### Laser Microdissection

The Leica^®^ laser microdissecting microscope (LMD6500) with LMD Software was used for microdissection. The 355 nm laser was set to isolate distinct tissues from the sectioned materials: the aleurone, subaleurone, and starchy endosperm were separated from the surrounding tissue by defining a closed cutting line, and the cut region was removed by gravity into a lid of a precooled PCR tube. Typically, aleurone and subaleurone were represented by 30 sections, in total equivalent to 2,100,000 μm^2^ of surface area. Similarly, the starchy endosperm was represented by ∼16 sections, equivalent in total to 6,300,000 μm^2^. Three independent preparations were processed per cell type for subsequent RT-qPCR.

### RNA Extraction and cDNA Synthesis

Barley seeds at 6, 10, 12, and ≥20 dap were harvested for subsequent gene and protein expression analyses. All samples were stored at -80°C until further processing. Total RNA was extracted from the WS as described in Li and Trick (2005). Total RNA from the LMD sections was extracted with a Picopure RNA isolation kit (Molecular Devices, Sunnyvale, CA, United States). We applied gene expression analysis by two step RT-qPCR according to the Minimum Information for Publication of Quantitative Real-Time PCR Experiments (MIQE) guidelines (Bustin et al., 2009). RNA concentration was measured at 260 nm and purity of RNA assessed by OD_260_/OD_280_ ratio using a UV spectrophotometer (NanoDrop Technologies, United States; **Supplementary Figures S2**, **S3**). RNA integrity of RNA isolated from WS as well as from different endosperm cell layers was assessed by a microfluidic capillary gel electrophoresis applying the Experion^TM^ system (Experion^TM^ RNA HighSens Analysis Kit, Bio-Rad Laboratories, United States) with Experion software 3.2P. The quality of RNA index (QRI) was between 4.7 (color indicator: yellow: possibly acceptable quality) and 9.7 (color indicator: green: acceptable quality) even though we are aware that plants have a further rRNA peak derived from 25S RNA. Selected RNA samples were stored at -80°C prior to use. DNA was digested from total RNA (10 ng/μL) with RNase-Free DNase Set (Qiagen, Germany). Prior to reverse transcription, samples were held at 42°C for 2 min with gDNA Wipeout Buffer (Qiagen, Germany). Reverse transcription was performed on 5 ng RNA in a final volume of 20 μL, using Quantitect Reverse Transcription Kit (Qiagen, Germany). Samples were incubated for 25 min at 42°C, 3 min at 95°C, and cooled at 4°C or stored at -80°C. cDNA synthesis and RT-qPCR were performed with three biological replicates. We used a pipetting robot (Qiagen, Germany) and RT-qPCR was performed with the Corbett Rotor-Gene Q (Australian TM Corbett Research Pty Ltd.) using the QuantiFast TM SYBR Green RT-PCR Kit (Qiagen, Germany). At least three pipetting replicates were for done for RT-qPCR. The PCR cycling conditions were set as follows: PCR initiation activation step for 5 min at 95°C, denaturation for 5 s at 95°C and combined annealing extension for 10 s at 60°C. The cycle number was 40. To verify the consistency of the amplicon, a melting point analysis was performed.

### RT-qPCR and Data Analyses

Besides bioinformatic analyses, the specificity of the primers was tested by PCR on cDNAs transcribed from RNAs extracted from WSs at 12 dap (**Supplementary Figure S2**). Quality analyses for all RNAs, RT-qPCRs’ parameters for each run and normalization was carried out for all WS and LMD analyses as well as for RGs and HINs studies and was performed as the following (**Supplementary Figures S2**–**S5**): performing RT-qPCR, a single peak was found for the melting curve for all 10 candidate RGs at each time point (data not shown). Slope, *R*^2^ value (squared correlation coefficient), and the PCR efficiency parameters were used to validate the PCR reaction of each primer pair. The amplification factor (AF) was calculated for each gene depending on the slope. Knowing that an optimal RT-qPCR reaction should have a slope of -3.32 (optimal range between -3.58 and -3.10), an efficiency of 100% and an *R*^2^ value of >0.99, we evaluated only the PCR runs closest to these values. To determine the relative quantities (*Q*), we calculated the mean Ct value for each biological replicate of four developmental stages for the WS analyses and of two developmental stages for LMD analyses. For the WS as well as for LMD analyses, *Q = E*^Δ^*^CT^*, where *E* is the efficiency of the gene amplification and Δ*CT* is the sample with the lowest Ct in the dataset minus the Ct value of the gene of interest and thus normalized to the lowest Ct value of the gene. The coefficient of variation (CV) was calculated for each candidate RG. The CV is the ratio of the standard deviation to the mean (average) of all Ct values of three biological replicates of a candidate RG. Thus, four representing values were used for the analysis using the statistical algorithms tool geNorm to calculate the expression stability value (*M*) for each candidate RG for the WS (Vandesompele et al., 2002; Andersen et al., 2004). For LMD studies, three values for 12 and ≥20 dap and six values for aleurone, subaleurone, and starchy endosperm were employed for the geNorm analyses. *M* describes the average of the pairwise variation of each particular gene with all other candidate reference genes. The lower the *M* value is, the more stably expressed the gene is. Additionally, the CV was calculated for each RG candidate to evaluate its expression stability.

For the normalization studies of *HIN* transcripts, we used the following RGs (**Supplementary Table S2**): *ARF*, *FBPA*, and *SAM* for WS; *GAP*, *GRP*, and *UBI* for LMD at 12 dap; *HSP90*, *ACPIIII*, and *ARF* for LMD at ≥20 dap; *HSP70*, *HSP90*, *GRP*, *ELF*, *UBI*, and FBPA for A (12 and ≥20 dap); *SAM*, *GRP*, *HSP70*, *ARF*, *HSP90*, *FBPA*, *ELF*, and *UBI* for SA (12 and ≥20 dap); and *ELF*, *FBPA*, and *UBI* for SE (12 and ≥20 dap). Normalization was calculated as described (Vandesompele et al., 2002). For statistical analyses, we performed a Student’s *t*-test [two-tailed distribution, two-sample unequal variance (heteroscedastic)] by the software Microsoft Excel.

### Sample Preparation for Proteomics Analyses, MS Measurement, and Data Analysis

Total proteins were extracted from barley seeds harvested at 6, 10, 12, and ≥20 dap as well as from LMD sections from seeds harvested at 12 and ≥20 dap in three biological replicates (**Supplementary Figure S1**) following an adapted protocol from (Roustan et al., 2017). A phenol-phase protocol extracted proteins. Samples were dissolved in a urea buffer and protein concentration was measured with a Bradford Assay prior to trypsin digestion. Following over-night digestion, peptides were desalted with C18 solid phase extraction (SPE) tips (Agilent Technologies, Santa Clara, CA, United States). After SPE, the corresponding eluates were dried in a vacuum concentrator. Peptide pellets were dissolved at a protein concentration equivalent to 0.1 μg/μL in 5% (v/v) ACN and 0.1% (v/v) formic acid (FA); 0.5 μg of the mixture was separated on an EASY-Spray PepMap RSLC 75 μm × 50 cm column (Thermo Fisher Scientific Inc., Waltham, United States). Peptides were eluted using a 240 min linear gradient from 2 to 40% of mobile phase B (mobile phase A: 0.1% [v/v] FA in water; mobile phase B: 0.1% [v/v] FA in 90% [v/v] ACN) with 300 nL/min flow rate generated with an UltiMate 3000 RSLCnano system. For the LMD samples, peptides were eluted with a 145 min linear gradient with the same mobile phase and percentage as for the WS proteomics. The peptides were measured with a LTQ-Orbitrap Elite (Thermo) using the following mass analyzer settings: ion transfer capillary temperature 275°C, full scan range 350–1800 *m/z*, FTMS resolution 120,000. Each FTMS full scan was followed by up to 10 data-dependent (DDA) CID tandem mass spectra (MS/MS spectra) in the linear triple quadrupole (LTQ) mass analyzer. Dynamic exclusion was enabled using list size 500 *m/z* values with exclusion width ± 10 ppm for 60 s. Charge state screening was enabled and unassigned and +1 charged ions were excluded from MS/MS acquisitions. For injection control, automatic gain control (AGC) for full scan acquisition in the Orbitrap was set to 5 × 105 ion population, the maximum injection time (max IT) was set to 200 ms. Orbitrap online calibration using internal lock mass calibration on *m/z* 371.10123 from polydimethylcyclosiloxane was used. Multistage activation was enabled with neural losses of 24.49, 32.66, 48.999, 97.97, 195.94, and 293.91 Da for the 10 most intense precursor ions. Prediction of ion injection time was enabled and the trap was set to gather 5 × 103 ions for up to 50 ms. Raw files were processed with MaxQuant 1.5^3^ and Andromeda search algorithm (Cox and Mann, 2008; Cox et al., 2011) on the *Hordeum vulgare sp. vulgare* Uniprot database (124,660 protein entries). Peptides identification was performed using the following settings: Mass tolerance for precursor was set to 5 ppm and for fragment masses up to 0.8 Da. The maximum FDR was set to 0.01%. Three missed cleavages were allowed. The dynamic modifications allowed were: methionine oxidation (M) and protein N-terminal acetylation. Carbamidomethyl (C) was allowed as fixed modification. Label-free quantification (LFQ) was performed based on at least two peptides per protein across the developmental stages and was internally normalized by MaxQuant (Cox et al., 2014). Quantification was calculated at peptide level. Further data processing (including one-way ANOVA) was performed with the Perseus 1.5 software (Tyanova et al., 2016). Annotated MS/MS spectra were visualized with MaxQuant (Tyanova et al., 2015). For statistical analyses, we performed a Student’s *t*-test [two-tailed distribution, two-sample unequal variance (heteroscedastic)] by the software Microsoft Excel. The mass spectrometry proteomics data have been deposited to the ProteomeXchange Consortium (Deutsch et al., 2017) via the PRIDE (Vizcaino et al., 2016) partner repository with the dataset identifier PXD009708 and PXD009722.

### Western Blot (WB) Protocol for HIN Detection

Developing seeds of barley were harvested at 6, 12, and ≥20 dap. One hundred milligrams of each fresh grain were squashed in 2 mL of reducing agent (25 mM Tris-HCl pH 7.8, 1.6% SDS, 100 mM DTT) using mortar and pestle on ice. The homogenate was centrifuged at 6800 *g* for 15 min at 4°C and the supernatant was transferred into a fresh microtube; 20 μL of SDS-PAGE sample buffer [containing 250 mM Tris-HCl pH 6.8, 10% (w/v) SDS, 0.5% (w/v) bromophenol blue and 50% (v/v) glycerol] was added to 80 μL of each sample, boiled for 5 min in microtubes, and subsequently cooled at room temperature. They were loaded in 5% stacking polyacrylamide gel and fractionated in 15% resolving gel for 2 h at a constant current of 25 mA under denaturing condition using a Bio-Rad mini-gel electrophoresis unit. The starting voltage was 52 V and final voltage 124 V. Electrode buffer was 25 mM Tris-base pH 8.8, 200 mM glycine, and 0.1% (w/v) SDS. After electrophoresis, the gel was presoaked in the blotting buffer [48 mM Tris-base pH 8.3, 39 mM glycine, 20% (v/v) methanol], together with 3 mm Whatman filter paper and nitrocellulose membrane for 30 min. Proteins were transformed from gel to nitrocellulose membrane using the Bio-Rad semi-dry transblotter at constant 18 V for 30 min. Following the transfer, the nitrocellulose membrane was blocked in 5% (w/v) non-fat powdered milk (1 h) prepared in a phosphate buffered saline including 0.1% Tween 20 (PBS-T pH 7.4). Immunoblots were incubated in 1:2000 dilution of anti-PINa/b rabbit antiserum prepared in PBS-T buffer for 2 h at room temperature or at 4°C shaking overnight, followed by washing three times in PBS-T, 5 min each. The second antibody was anti-rabbit IgG–alkaline phosphatase conjugate that was diluted 1:5000 and incubated for 1 h. Signal detection was performed by ready-to-use reagents (Bio-Rad).

### Fixation, Semi-thin Sectioning, and Immunofluorescence

At least three randomly selected seeds were harvested from the mid-section of the ear at 12 and ≥20 dap (**Supplementary Figure S1**). They were tangentially and vertically cut into one to 2 mm^2^ pieces and fixed in 2.5% paraformaldehyde and 0.25% glutaraldehyde in 0.1 M phosphate buffer (PB) pH 7.4 at 4°C overnight. The chemically fixed samples underwent low-temperature dehydration and infiltration with methacrylic resin, Lowicryl HM20 (Polysciences, Warrington, PA, United States), by applying the progressive lowering of temperature technique (Carlemalm et al., 1985). After washing in PBS, the samples were dehydrated in a series of ethanol (30% ethanol for 30 min on ice; 50% ethanol for 45 min, two times 70% ethanol for 30 min each, 95% ethanol for 45 min, and two times 100% ethanol for 30 min each, on salt ice at -20°C). Subsequently, samples were infiltrated with HM20 at -20°C (1/3 volume HM20 and 2/3 volume ethanol for 1 h, 2/3 volume HM20 and 1/3 volume ethanol for 2 h, pure HM20 for 1 h). The infiltration with pure resin was continued for further 6 h at -40°C in an automated freeze substitution unit (AFS2, LEICA Microsystems, Austria). For UV polymerization, samples were transferred into PCR tubes, Multiply^®^ 0.2 mL (Sarstedt) that were attached to spider covers (LEICA Microsystems, Austria) as described previously (Reipert and Wiche, 2008). UV-polymerization was performed for 36 h at -40°C.

Semithin sections (1.5 μm) of the seeds were cut by an ultramicrotome LEICA EM UC7 (Wetzlar, Germany) and a semi-diamond knife (DIATOME Ltd., Switzerland), collected on a glass slide and dried at 40°C. Seed sections were rinsed in PBS for 20 min and blocked with 5% BSA in 0.1 M PB, for 15 min at room temperature. The sections were incubated with rabbit polyclonal anti-PINa/b (diluted 1:50 in 0.1 M PB) followed by three times washing by 0.5% Tween in 0.1 M PB. The secondary antibody Alexa488^®^ was diluted 1:30 in 0.1 M PB and incubated for 1 h at room temperature. Sections were washed in 0.1 M PB and distilled water three times for 10 min each, followed by air drying. At least two slides containing three sections each were analyzed and images were quantified with ImageJ. For statistical analyses, we performed a Student’s *t*-test ([two-tailed distribution, two-sample unequal variance (heteroscedastic)] by the software Microsoft Excel.

## Results

### Detection and Quantification of *HIN* Transcripts in WS During Barley Endosperm Development

**FIGURE 1:**
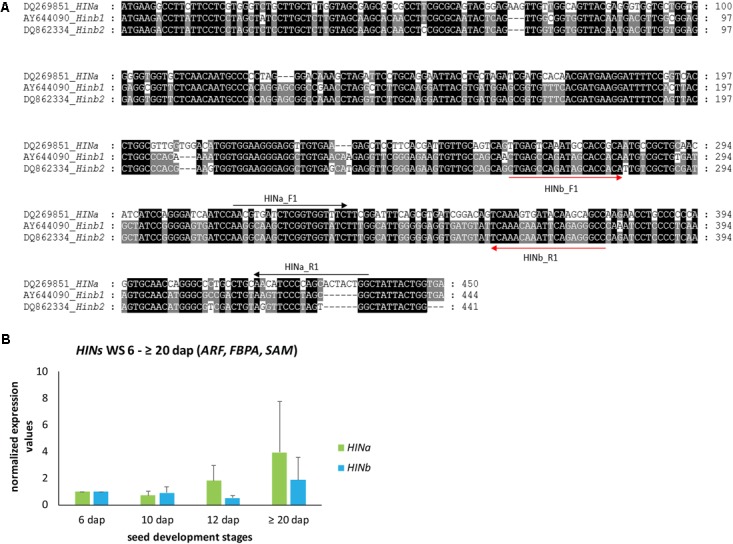
Gene alignment and quantification of *HINa* and *HINb* transcripts in WS during barley endosperm development. **(A)** cDNA alignment of *HINa*, *Hinb1*, and *HINb2* performed by MEGA7.0.21 (Kumar et al., 2016) and visualized by GenDoc (Nicholas and Nicholas, 1997). Conserved percentage is shown as the following: black 100%, dark gray 80%. **(B)** Bar graph describes the average over three biological replicates of the normalized transcripts from *HINa* and *HINb* at 6, 10, 12, and ≥20 dap with the most stable RGs (*ARF*, *FBPA*, and *SAM*). Bars represent standard deviation. For statistical analyses, we performed a Student’s *t*-test. Note the trend of all *HIN* transcripts to increase between 6 and ≥20 dap.

In order to explore the dynamics of *HIN* transcripts during endosperm development, we analyzed first the levels of *HINa* and both *HINb* transcripts in developing WS. cDNA alignment analyses of *HINa*, *HINb1*, and *HINb2* showed that the DNA sequence *HINa* is different to *HINb1* and *HINb2*, but the cDNA sequences of *HINb1* and *HINb2* are very similar (**Figure 1A**). Thus, specific primers could only be designed for *HINa* and *HINb* resulting in small PCR products (**Figure 1A**, **Supplementary Figure S6**, and **Supplementary Table S3**). According to our geNorm results, we used the three most stable RGs *ARF*, *FBPA*, and *SAM* to normalize *HIN* transcripts in WS during endosperm development at 6, 10, 12, and ≥20 dap. No significant changes of the abundance of *HIN* transcripts could be observed in WSs during development, even though both *HIN* transcripts showed a trend to increase during development, especially the *HINa* transcripts (**Figure 1B**). These results correlate to data previously shown where *HIN* transcripts increased during late development stages (Darlington et al., 2001). To confirm the stability of the selected RGs, we normalized the *HIN* transcripts to the lowest stable RG *GRP*. The bar chart shows a decrease for both *HIN* transcripts between 6 and ≥20 dap and subsequently no reliable normalization results (**Supplementary Figure S7**).

### Detection and Quantification of HIN Proteins in WS During Barley Endosperm Development

**FIGURE 2:**
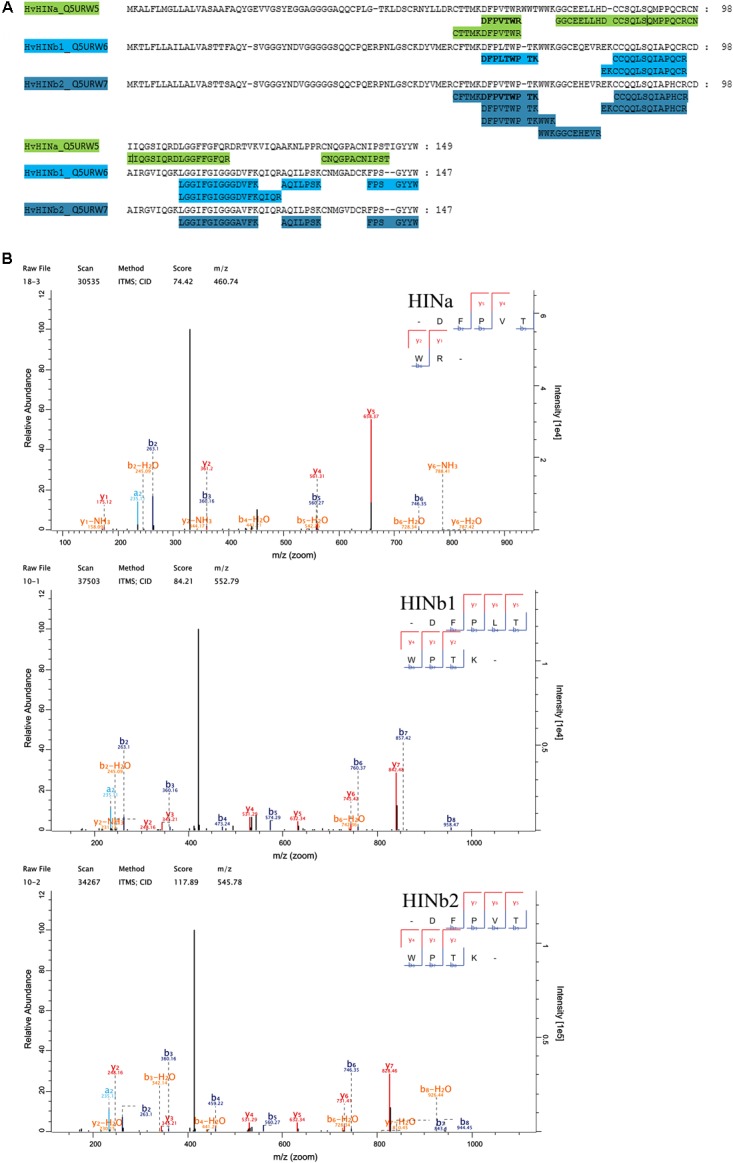
Proteomic identification of HIN proteins. **(A)** Identified peptides in the proteomic approach have been aligned with HINa, HINb1, and HINb2 protein sequence. **(B)** MS/MS spectra of the identified tryptophan-rich peptides of HINa, HINb1, and HINb2.

**FIGURE 3:**
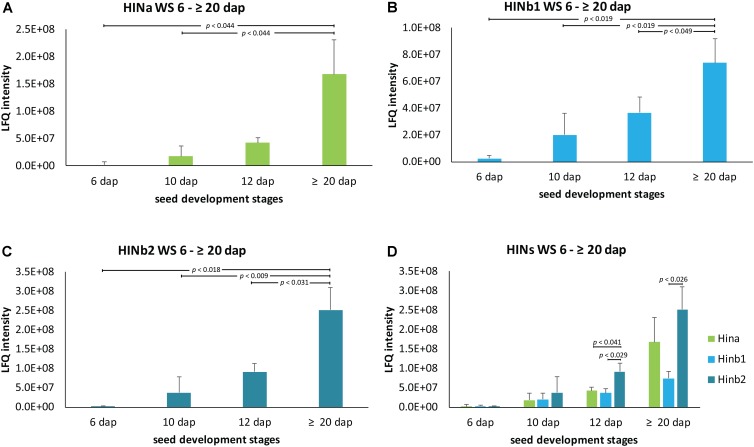
LFQ quantification of HIN proteins in WS between 6 and ≥20 dap based on ion precursor intensity. **(A)** HINa, **(B)** HINb1, and **(C)** HINb2 protein abundance in WS between 6 and ≥20 dap. **(D)** Comparative analysis of HINa, HINb1, and HINb2 abundance. For statistical analyses, we performed a Student’s *t*-test. LFQ intensities of proteins were averaged over three biological replicates. Bars represent standard deviation. Note the indicated *p*-values.

Quantitative proteomics across barley endosperm developmental stages was performed. In total, 1029 proteins were quantified across the four studied stages of barley grain filling in all samples (**Supplementary Table S4**). At the protein level, MS1 precursors were used to specifically quantify HINa as well as HINb1 and HINb2. In total, two peptides were common to HINb1 and HINb2 and seven, four, and six peptides were unique for HINb2, HINb1, and HINa, respectively (**Figure 2A** and **Supplementary Figure S8A**). As well, some of the peptides were found in the LMD-based proteomics approach as indicated in **Supplementary Figure S8A**. Mass spectrometry analysis specifically identified proteotypic peptides corresponding to the tryptophan-rich domain of HINa, HINb1, and HINb2 (**Figure 2B** and **Supplementary Figure S8B**) by the presence of three trypsin-target residues (R/K). In barley, the tryptophan-rich domain of HINb2, 61 – KDFPVTWPTKWWKG – 74, presented exactly the same amino acid sequence as PINb in wheat (Wall et al., 2010). Quantification of the HIN proteins at the WS level highlights a constant significant increase of the HIN abundance from 6 to ≥20 dap (**Figures 3A–C**). Between 6 and ≥20 dap, HINb2 became the most abundant protein (**Figure 3D**). More specifically, HINb2 was approximatively 1.5 times more abundant than HINa and 3.4 times more abundant than HINb1 at ≥20 dap (**Figure 3D**). Those proportions are in line with the HIN proportion found previously in mature seeds (Mahalingam, 2017). To confirm the LC-MS data, we performed a western blot (WB) of protein extracts of 6, 12, and ≥20 dap. HINs are barley homologs of wheat PIN and oat TRP proteins that show high similarity at the level of amino acid sequence (**Supplementary Figure S8B**) confirmed also by MS/MS spectrum (**Figure 2B**). Therefore, we used anti-PINa/b antisera (kindly provided by Illimar Altosaar) that recognize both A- and B-isomers of the wheat PIN homolog (Mohammadi et al., 2006). Whereas hardly any band could be detected at 6 and 12 dap, anti-PINa/b recognized two bands at ≥20 dap (**Supplementary Figure S8C**). As the full protein of HINa has 16.4 kDa and HINb1/b2 16.1/16 kDa, we concluded that the upper band represents HINa and HINb proteins. The lowest band described a 13 kDa protein, which represents the mature protein without signal peptide and propeptides. This indicates a specific recognition of barley HINs by anti-PINa/b, as the WB showed no signal when incubated by the pre-immune-serum (**Supplementary Figure S8C**). Thus, HINs are most accumulated at ≥20 dap where HINb2 describes the most abundant protein, followed by HINa and finally HINb1.

### HINs Are Mainly Localized at PSVs in Aleurone, at PBs in Subaleurone, and at the Periphery of Starch Granules in the Starchy Endosperm

**FIGURE 4:**
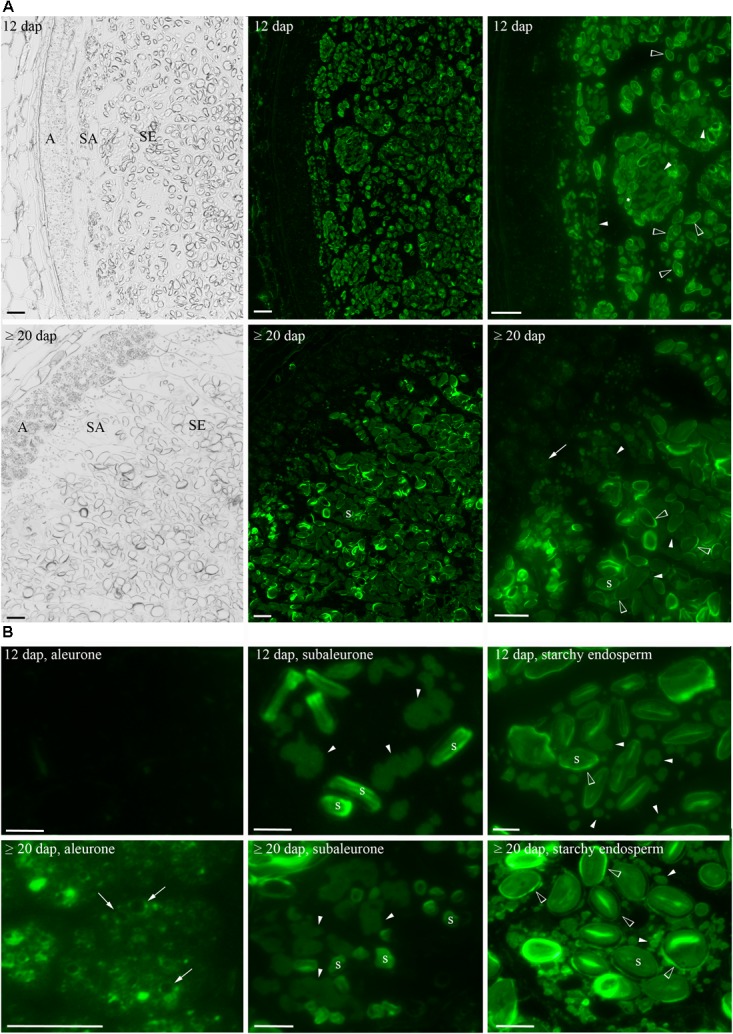
Spatio-temporal localization of HINs during barley endosperm development. **(A)** Anti-PINa/b labeling of HINs at 12 and ≥20 dap. Note the layers of aleurone (A), subaleurone (SA), and the starchy endosperm (SE) in the bright field channel. At 12 dap, no signal of HINs could be detected in aleurone, whereas a faint signal could be detected in A (arrows) at ≥20 dap. At 12 and ≥20 dap, anti-PINa/b strongly labels protein bodies (PBs) in SA and in SE, respectively (arrowheads). **(B)** Close-up of a cell representing each tissue. The signal at PBs appears to be constant in SA between 12 and ≥20 dap (arrowheads). Note the signal of HINs at the vacuolar membrane (arrows) in the A at ≥20 dap and the strong signal at the periphery of starch granules in the SE at ≥ 20 dap (open arrowheads). Labeled PBs show reduced sizes in SE at 12 and ≥20 dap (arrowheads). Scale = 100 μm. s indicates starch granules.

As HINs start to accumulate at 12 dap and increased to ≥20 dap, these two timepoints were chosen to explore the localization of HINs in developing barley grain. Seeds were harvested at 12 and ≥20 dap; fixed and semi-thin sections (1.5 μm) were prepared for microscopic studies. We used anti-PINa/b antibodies for a spatio-temporal visualization of both HIN isoforms (a and b) by immunofluorescence. We could observe a strong signal in the subaleurone and starchy endosperm at 12 dap as well as at ≥20 dap (**Figure 4A**), whereas no signal was observed in the negative control (**Supplementary Figure S9**). Using a higher magnification, we could identify a prominent fluorescent signal at the typically shaped PBs (Ibl et al., 2014) and at the periphery of starch granules in subaleurone at 12 and ≥20 dap (**Figure 4B**). The signal at the PBs in subaleurone appeared to remain constant, whereas the amount of intensively labeled PBs increased in the starchy endosperm between 12 and ≥20 dap (**Figure 4B**). The signal at the periphery of starch granules was detectable in the starchy endosperm at 12 dap and became stronger at ≥20 dap. Interestingly, a weak signal was observed at the PSV membrane in the aleurone tissue at ≥20 dap (**Figure 4B**). We quantified the fluorescent signal of several sections in aleurone, subaleurone, and starchy endosperm at 12 and ≥20 dap by ImageJ (**Supplementary Figure S10A**; Schindelin et al., 2012; Schneider et al., 2012). At 12 dap, the signal in subaleurone and starchy endosperm was 2.7 and 4.8 times stronger than in aleurone, respectively (**Supplementary Figure S10B**). The signal at ≥20 dap was again stronger in starchy endosperm (two times more) and in subaleurone (1.2 times more) than in aleurone. Within one tissue, the strongest increase of the fluorescent signal between 12 and ≥20 dap could be observed in aleurone (three times more), followed by the increase of the signal in the starchy endosperm (1.4 times more). These results strongly point to a spatio-temporal increase of the HIN protein abundance in barley endosperm. Additionally, we observed subcellular localization alterations of HINs depending on the cell layer and development stage. Especially the high HIN abundance in the starchy endosperm at 12 and ≥20 dap is accompanied by an accumulation of HINs at the periphery of starch granules.

### Quantification of Spatio-Temporal HIN Proteins and *HIN* Transcripts in Developing Barley Endosperm

**FIGURE 5:**
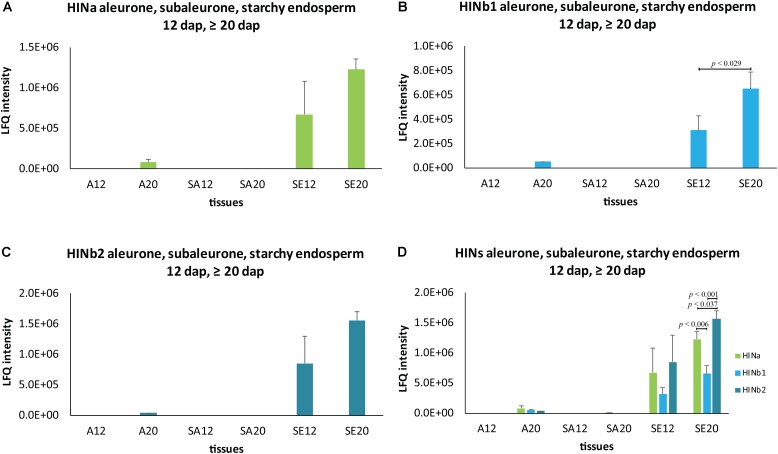
Spatio-temporal HIN protein LFQ quantification in developing barley endosperm. Protein quantification of **(A)** HINa, **(B)** HINb1, and **(C)** HINb2 in aleurone (A), subaleurone (SA), and in starchy endosperm (SE) at 12 and ≥20 dap. **(D)** Overview of HIN proteins in A, SA, and SE at 12 and ≥20 dap. For statistical analyses, we performed a Student’s *t*-test. LFQ intensities of proteins were averaged over three biological replicates. Bars represent standard deviation. Note the indicated *p*-values.

To get further insight into the proportion of HIN proteins and *HIN* transcripts in aleurone, subaleurone, and starchy endosperm during the grain filling process, we used LMD for sampling three different tissues at two timepoints (12 and ≥20 dap) for subsequent quantitative shotgun proteomics and RT-qPCR. As indicated by the immunolabeling of the HIN proteins, there is an important dynamic range between the starchy endosperm and the aleurone and subaleurone layer. Such a dynamic range affects the peptide detection, since in mass spectrometry, there is no amplification of the signal such as in RT-qPCR. This effect was supported by the LMD-based shotgun proteomics data. Indeed, most of the peptides’ identification and quantification were performed in the starchy endosperm tissue (**Supplementary Table S4**). More precisely, the three HIN proteins were well detectable in the starchy endosperm at both 12 and ≥20 dap (with a consistent quantification of at least two peptides per proteins). The identification rate is less in the aleurone and subaleurone layer: only one peptide specific to HINb2 was detectable in the aleurone layer at ≥20 dap. This is in line with the detected increase of HIN content in the aleurone cell layer between 12 and ≥20 dap by immunofluorescence (**Supplementary Figure S10**). The increase of HIN proteins in aleurone from 12 to ≥20 dap is further supported by the HINa and HINb1 peptides detection in only one sample in aleurone tissue at ≥20 dap (**Supplementary Table S4**). Surprisingly, a single peptide belonging to HINa was detected in one subaleurone sample at ≥20 dap. In order to approximate a distribution pattern of the HINs, we averaged the intensity of the peptides and plotted them in a spatio-temporal pattern (**Figure 5** and **Supplementary Table S4**). Whereas HINa and HINb2 showed the trend to increase in the starchy endosperm between 12 and ≥20 dap (**Figures 5A,C**), HINb1 increased significantly (**Figure 5B**). HINb2 is the most abundant HIN protein in starchy endosperm at ≥20 dap, followed by HINa and finally HINb (**Figure 5D**). These results confirmed the major contribution of the starchy endosperm to the total seed HIN content (**Figure 5D**). Interestingly, the proportions between the HIN protein abundances are possibly conserved in the starchy endosperm between 12 and ≥20 dap as the same distribution of HIN proteins could be detected.

**FIGURE 6:**
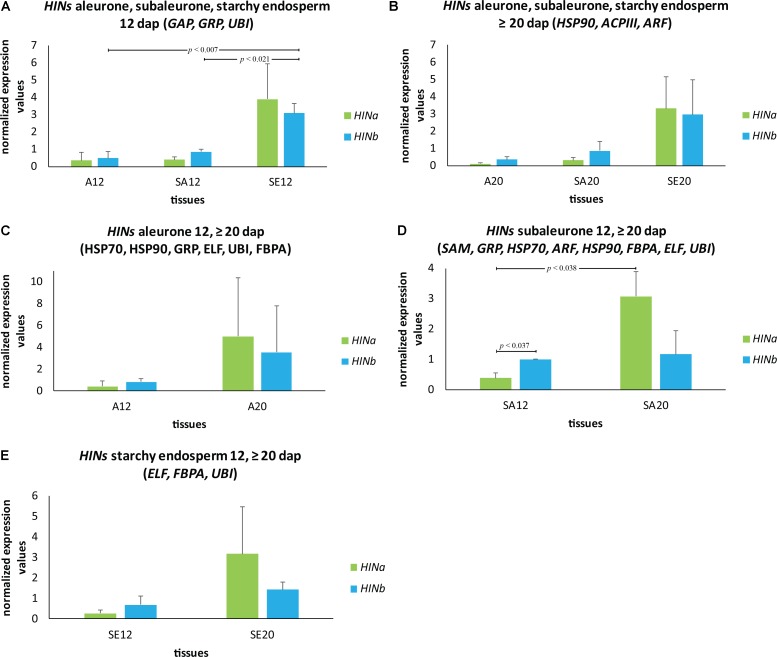
Spatio-temporal *HIN* transcripts quantification in developing barley endosperm. Bar graph describes the average over three biological replicates of the normalized transcripts from *HIN* transcripts in aleurone (A), subaleurone (SA), and starchy endosperm (SE) at **(A)** 12 and **(B)** ≥20 dap. HIN transcripts in **(C)** A, **(D)** SA, and **(E)** SE at 12 and ≥20 dap. For statistical analyses, we performed a Student’s *t*-test. Bars represent standard deviation. Note the indicated *p*-values.

To get a complete spatio-temporal RNA expression overview of the *HIN* transcripts, we quantified changes in the abundance of *HIN* transcripts in aleurone, subaleurone, and starchy endosperm during development by using our characterized RGs for these tissues (**Figures 6A–E**). At 12 dap, *HINb* transcripts were significantly more abundant in subaleurone and starchy endosperm compared to aleurone (**Figure 6A**). The most significant changes of *HINa* transcripts could be observed in subaleurone (**Figure 6D**), where they increased significantly between 12 and ≥20 dap (**Figure 6D**). Interestingly, *HINb* transcripts are more abundant than *HINa* ones in subaleurone at 12 dap (**Figure 6D**), whereas only HINa was detectable in subaleurone at ≥20 dap.

These data suggest a spatio-temporal regulation of both transcription and translation of HINs where the subaleurone showed a poor correlation between HIN transcripts and proteins.

## Discussion

Barley grain ranks fourth in cereal production worldwide but is the second largest cereal crop within the European Union (19.5 % of total cereal production^4^). Besides its importance as food and feed source, the most frequent use of barley is for malting purposes for the brewing industry (Gupta et al., 2010). Grain texture of barley has a huge impact on the malting performance, and soft barley cultivars have better malting quality (Brennan et al., 1996; Psota et al., 2007; Gupta et al., 2010). The endosperm-specific PIN orthologs HINa, HINb1, and HINb2 are associated with both major endosperm texture and grain hardness in barley.

**FIGURE 7:**
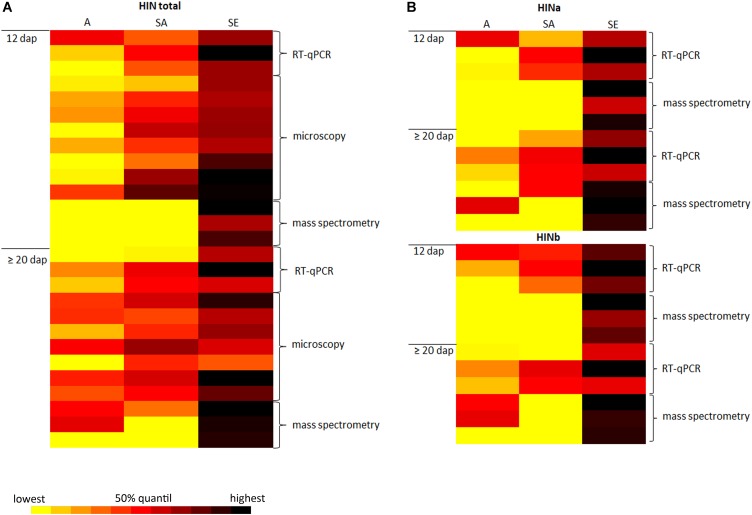
Data-matrix heat map illustrating the correlation of RT-qPCR, microscopy, and mass spectrometry data of the spatio-temporal expression abundance of HINs in developing barley endosperm. **(A)** Data-matrix heat map of all HINs. RT-qPCR cluster represents the normalization results (*n* = 3), the microscopy data represent the area calculation (*n* = 8 for 12 dap; *n* = 7 for ≥20 dap), and the mass spectrometry data represent the total amount of HINs (*n* = 3). **(B)** Data-matrix heat map of HINa and HINb. RT-qPCR cluster represents the normalization results (*n* = 3) of *HINa* and *HINb*, and the mass spectrometry data represent the total amount of HINa (*n* = 3) and HINb (*n* = 3). Heat map was prepared by the Software Microsoft Excel. Scale: yellow = smallest value; red = 50% quantil; black = highest value.

In wheat, PINa and PINb proteins are associated with grain hardness and endosperm/kernel texture, the leading characteristics of common “bread” wheat. In addition, PINs are important players concerning the foam formation and stability of beer as the addition of PIN proteins to degas could protect beer foam against lipid-induced destabilization (Bamforth, 1985; Clark et al., 1994). PINs are furthermore involved in antimicrobial activities and therefore considered for developing applications (reviewed in Bhave and Morris, 2008). While the spatio-temporal regulation of PINs expression is quite well studied in wheat, little is known about their homologous proteins in barley. Therefore, it is essential to understand the molecular regulation of HIN proteins during barley endosperm development. For this, a detailed analysis of the spatio-temporal expression of HINs during barley endosperm development as well as their subcellular localization is necessary. In this regard, we conduct three main experiments: first, we identified RGs for reliable quantification studies of *HIN* transcripts during WS endosperm development as well as in three different tissues at two different time points. Second, we performed proteomics to quantify the protein abundances of HINa, HINb1, and HINb2 during WS endosperm development as well as in each cell layer at different timepoints. Third, we studied the localization of HIN proteins during barley endosperm development. The combination of RT-qPCR, proteomics, and microscopic analyses of HINs not only gives insight into the spatio-temporal regulation during barley development, but also strengthens our data as the results are correlating. This is illustrated by the data-matrix heat map in **Figure 7** that shows a high abundance of all *HIN* transcripts and HIN proteins in starchy endosperm at ≥20 dap.

**FIGURE 8:**
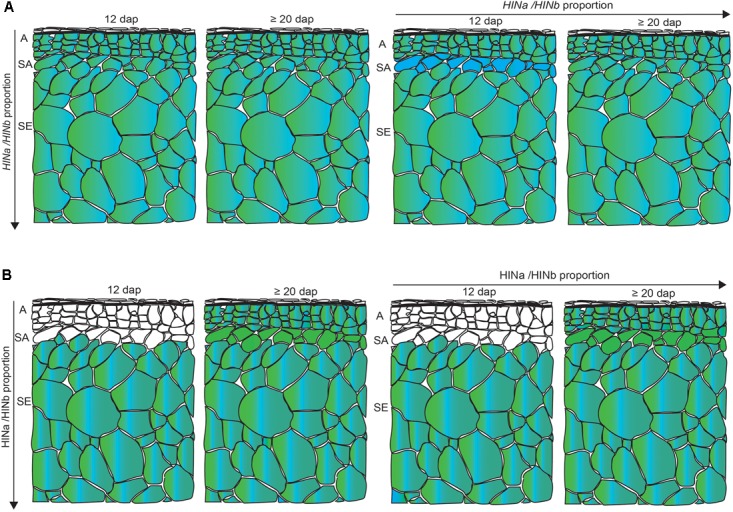
Schematic overview of the spatio-temporal *HIN* transcript and HIN protein abundance in developing barley endosperm. **(A)** *HINa/HINb* transcripts proportion in all tissues between 12 and ≥20 dap and between aleurone (A), subaleurone (SA), and starchy endosperm (SE) between 12 and ≥20 dap. **(B)** HINa/HINb protein abundance proportion in all tissues between 12 and ≥20 dap and between A, SA, and SE between 12 and ≥20 dap. Color code: green, HINa; bright blue, HINb1; and turquoise, HINb2. White cells represent no detected HIN proteins. Note the differences in the SA layer. Figure was drawn by the software program Adobe Illustrator CC 2015.

Having developed a new panel of RGs for spatio-temporal normalization studies in barley GP, we quantified the transcripts of *HINa* and *HINb* in the WS at 6, 10, 12, and ≥20 dap. We could not separate *HINb1* from *HINb2* by RT-qPCR as no specific primer could be designed due to their overlapping cDNA sequence. Both *HINa* and *HINb* transcripts showed the trend to increase at ≥20 dap. Using LC-MS and WB analyses, we could detect an increase of the protein abundance for both HINa and HINb from 6 to ≥20 dap, indicating a correlation between the amount of *HIN* transcript and protein abundance. Although the HINbs are very similar proteins, the resolution of LC-MS allowed the identification of unique peptides for both HINb1 and HINb2: out of the 19 identified peptides, 17 were unique peptides allowing a reliable identification and quantification of HINa, HINb1, and HINb2. To analyze the *HIN* transcript as well as the protein abundance in more detail, we performed spatio-temporal RT-qPCR as well as proteomics to quantify the transcript and protein levels at 12 and ≥20 dap. At both 12 and ≥20 dap, the amounts of *HIN* transcripts and HIN proteins were higher in starchy endosperm than in aleurone and subaleurone. However, *HINb* transcripts were highly abundant in subaleurone at 12 dap where no HIN proteins could be detected. Additionally, whereas *HINa* transcripts increased significantly in subaleurone between 12 and ≥20 dap, there is no significant difference between the final amount of *HINa* and *HINb* transcripts in subaleurone at ≥20 dap. At the protein level, only one peptide of HINa could be detected in subaleurone at ≥20 dap, indicating that HINa is more abundant than HINbs. These results point to an absence of the correlation between the *HIN* transcripts and HIN protein abundances in the subaleurone (**Figure 8**). This difference between transcript and protein levels was already described and discussed during maize endosperm development (Walley et al., 2013). In this context, it would be interesting to assess the stability and the translation rate of the *HIN* transcripts in all tissues, especially in subaleurone. Similarly, a comparative study between the different HIN protein stabilities could be conducted.

The presence of HINs was studied previously on molecular level but no information is available about their subcellular localization. PINs in wheat as well as TRPs in oat are known to accumulate at starch granules in late-developed and mature cells (Dubreil et al., 1998; Mohammadi et al., 2006). The used polyclonal anti-PINa recognized both PINs in immunostaining studies in aleurone and subaleurone, whereas monoclonal anti-PINa antibody only gave signals in the starchy endosperm, suggesting a different spatial distribution (Dubreil et al., 1998). Both PINs PINa and PINb were detected previously at PBs in developing wheat endosperm, describing the same trafficking pathway as prolamins from the endoplasmic reticulum (ER) to PBs (Lesage et al., 2011; Ibl and Stoger, 2012). As PINs show a close structural relation to 2S storage protein, it was suggested that PINs could be 2S-like storage proteins in wheat and possibly interact with prolamins via their tryptophan-rich domain (Lesage et al., 2011). This result points to a link between endosperm texture and storage protein aggregation. Here, we showed that the localization of HIN proteins is spatio-temporally regulated within the barley endosperm as we found different strong fluorescent signals at PBs and at the periphery of starch granules at 12 and ≥20 dap. At 12 and ≥20 dap, we detected HINs mainly in the subaleurone and in the starchy endosperm at PBs and additionally at the periphery of starch granules. At ≥20 dap, the fluorescent signal around the starch granules increased while the signal in the PB remained stable in the starchy endosperm. An additional signal was visible in the aleurone at ≥20 dap, indicating the presence of additional HIN proteins at ≥20 dap in the aleurone. As we have been working with polyclonal anti-PINa/b, we were not able to discriminate between HINa and HINb proteins in these localization studies.

Different final localizations of HINs may include the involvement of diverse protein trafficking pathways. However, the precise trafficking route of HINs to their final destinations is still unclear. The cereal endosperm contains specialized organelles for the accumulation of SSP, which are ultimately deposited either within PB derived from the ER, or in PSVs (reviewed in Arcalis et al., 2014). Besides dense vesicles, multivesicular bodies (MVBs) thereby play a critical role in post-Golgi transport of SSP toward the vacuole (reviewed in Ibl and Stoger, 2012). Additionally, an unusual autophagy-like mechanism for the vacuolar delivery of prolamins was discussed in maize aleurone cells where prolamin-containing compartments are eventually fusing with MVBs to form hybrid prevacuolar compartments (Reyes et al., 2011). Obviously, SSP transport routes depend on the cereal species, endosperm tissue layer, and developmental timepoint (Ibl and Stoger, 2012; Zheng and Wang, 2014). Recently, we showed that the involvement of HvVPS60a, a component of the ESCRT machinery coordinating the sorting of ubiquitinated membrane proteins into intraluminal vesicles (ILVs), is depending on the cell layer (Winter and Hauser, 2006; Hilscher et al., 2015). Additionally, OsVPS22 (ESCRT-II) is supposed to be required for seedling viability and grain filling in rice as the *vps22* mutant in rice endosperm showed a chalky endosperm (Zhang et al., 2013). We identified HINs as putative weak interaction partners of an ESCRT-III protein by co-immunoprecipitation and yeast two-hybrid studies (manuscript in preparation). These results let us speculate about the involvement of the ESCRT machinery in the targeting of HINs in barley endosperm development. Further investigations are needed in barley endosperm to follow the trafficking dynamic of HINs.

Finally, we propose another explanation for the different localization of HIN proteins observed in our immunofluorescent microscopy results: indeed, the identified peptides indicate that trypsin digestion can release cytotoxic peptides. The cytotoxicity of tryptophan-rich peptides has been studied and it is suggested that the cytotoxic mechanism seems to affect membrane permeability (Alfred et al., 2013; Haney et al., 2013; Ishida et al., 2016; Shagaghi et al., 2016). Is it legitimate to interrogate the cytotoxicity of tryptophan-rich peptides for cereal cells? And if yes, what is the scavenging process? In this context, it is worth to mention that PINs are still present in baked and stored food products (Capparelli et al., 2005; Pauly et al., 2013a,b).

Indeed, the question arises how the spatio-temporal regulated HIN protein abundance and HINs’ localization affect the food quality? The differences in the endosperm texture related to hardness have an impact on the properties and quality of flour: soft wheat flour is used for producing cake and cookies hard wheat flour for bread (summarized in Pauly et al., 2013a,b). However, it is still unclear how PINs behave in the cereal-based end-products, as most of the lipid binding capacity and foaming properties have been demonstrated *in vitro.* Nevertheless, previous studies showed that the amount of PINb bound to starch in soft wheat is twofold higher than that of HINa and HINb in barley cultivars (Gazza et al., 2008; Galassi et al., 2012). However, Galassi et al. (2012) suggested that the ratio between HINb/HINa is important for the hard texture of the barley grain as the PINb/PINa ratio in soft-textured wheat is threefold higher than the HINb/HINa ration in barley. It was further speculated that the poor interaction of HINa and HINb during their deposition on the starch surface is responsible for the different barley kernel texture (Galassi et al., 2012). Our results bring evidence that at ≥20 dap, the protein abundance differs significantly between HINa and HINb1/HINb2 in starchy endosperm but not at 12 dap. Additionally, HINa transcripts as well as HINa proteins accumulate specifically in the subaleurone at ≥20 dap, pointing to a different ration of HINb/HINa in this tissue and thus subsequently indicating a putative additional function of HINa in subaleurone. Therefore, it would be necessary to study the localization with specific antibodies for HINa and HINb to analyze the HINb/HINa ratio on subcellular level and to elucidate the spatio-temporal protein trafficking pathway necessary for their final destinations in barley endosperm.

## Conclusion

In this study, we used RT-qPCR, proteomics, and microscopy to unravel the spatio-temporal HIN expression and subcellular localization alterations in developing GP endosperm. To enable RT-qPCR in the cultivar GP, we characterized RGs for spatio-temporal normalization that can be used to carry out gene transcript analyses in developing GP endosperm. Based on our correlating results, we show that the expression abundance of HINs and the ration of HINb/HINa is tissue dependent during GP endosperm development. These findings may point to the necessity to study cereal endosperms tissue specific to improve plant crop engineering in terms of grain kernel texture modifications and end-product quality. Therefore, a good transcriptomic and proteomic map will help to understand the molecular mechanism behind these rearrangements. This will subsequently enable to specifically manipulate the HINb/HINa ratio in different tissues during development stages that could result in hard or soft barley kernel texture and thus influences the end-product quality.

## Author Contributions

VI acquired funding. VI and VR conceived and designed the experiments. AS, VR, and VI wrote the manuscript. AS performed the RT-qPCR analyses. VR did the MS analyses. P-JR performed the western blots. SR did the embedding. MW performed the semi-thin sectioning. ES and WW contributed toward the data analyses. All authors read and approved the final version of the manuscript for publication.

## Conflict of Interest Statement

The authors declare that the research was conducted in the absence of any commercial or financial relationships that could be construed as a potential conflict of interest.

## Footnotes

1 http://webblast.ipk-gatersleben.de/barley_ibsc/

2 http://www.uniprot.org/

3 http://www.maxquant.org

4 http://ec.europa.eu/eurostat/

## Supplementary Material

FIGURE S1 Schematic workflow of our multi-disciplinary approach as described in Section “Materials and Methods.”

FIGURE S2 RT-qPCR reaction quality control and amplification efficiency of 10 candidate RGs for RT-qPCR in the barley cultivar GP during endosperm development. **(A)** Detection of cDNA of all RGs. M: DNA ladder 100 bp. Primer pairs are indicated by an asterisk. **(B)** Quantification of RNA extracted from seeds at 6, 10, 12, and ≥20 dap. RNA integrity was controlled photometrically (260/280). **(C)** Results from standard curves of the selected candidate RGs: slope, *R*^2^ value, reaction efficiency (RE), and amplification factor (AF). **(D)** Relative quantification of all RGs from the whole seed harvested at 6, 10, 12, and ≥20 dap. **(E)** Coefficient of variation (CV) of all RGs. **(F)** Average expression stability (*M*) of all RGs from the whole seed harvested at 6, 10, 12, and ≥20 dap calculated by geNorm. **(G)** Calculation of the pairwise variation by geNorm.

FIGURE S3 RT-qPCR reaction quality control and amplification efficiency of 10 candidate RGs for spatio-temporal RT-qPCR during GP endosperm development. **(A)** Quantification of RNA extracted from aleurone, subaleurone, and starchy endosperm at 12 and ≥20 dap. RNA integrity was controlled photometrically (260/280). **(B)** Results from standard curves of the selected candidate RGs: slope, *R*^2^ value, reaction efficiency (RE), and amplification factor (AF). **(C)** Relative quantification of all RGs from all tissues at 12 and ≥20 dap. **(D)** Relative quantification of all RGs from and from aleurone (A), subaleurone (SA), and starchy endosperm (SE) at 12 and ≥20 dap.

FIGURE S4 RT-qPCR reaction quality control and amplification efficiency of 10 candidate RGs for RT-qPCR in GP endosperm development at 12 and ≥20 dap for all tissues. **(A)** Coefficient of variation (CV) of all RGs for all tissues at 12 and ≥20 dap. **(B)** Average expression stability (*M*) of all RGs from the all tissues at 12 dap calculated by geNorm. Note the calculation of the pairwise variation by geNorm. **(C)** Average expression stability (*M*) of all RGs from the all tissues at ≥20 dap calculated by geNorm. Note the calculation of the pairwise variation by geNorm.

FIGURE S5 RT-qPCR reaction quality control and amplification efficiency of 10 candidate RGs for RT-qPCR in barley endosperm for aleurone (A), subaleurone (SA), and starchy endosperm (SE) at 12 and ≥20 dap. **(A)** Coefficient of variation (CV) of all RGs for A (12 dap, ≥ 20 dap), SA (12 and ≥20 dap), and SE (12 and ≥20 dap). Average expression stability (*M*) of all RGs for **(B)** A (12 and ≥20 dap), **(C)** SA (12 and ≥20 dap), and **(D)** SE (12 and ≥20 dap). Note the calculation of the pairwise variation by geNorm.

FIGURE S6 RT-qPCR reaction quality control and amplification efficiency of the *HIN* genes by RT-qPCR in barley endosperm. **(A)** cDNA amplification of *HINa* and *HINb*. M: DNA ladder 100 bp. **(B)** Results from standard curves of *HINa* and *HINb*: slope, *R*^2^ value, reaction efficiency (RE), and amplification factor (AF).

FIGURE S7 Bar graph describes the average over three biological replicates of the normalized transcripts from *HINa* and *HINb* at 6, 10, 12, and ≥20 dap with the most unstable RG (*GRP*). Bars represent standard deviation.

FIGURE S8 Identification and molecular characterization of HINa, HINb, and HINb2. **(A)** Identified peptides related to HINa (green), HINb1 (bright blue), and HINb2 (turquoise). **(B)** Protein alignment of the tryptophanins (oat), puroindolines (wheat), and hordoindolines (barley). Note the signal peptide, propeptide, the tryptophan-rich side, and the cysteine amino acids (orange box). Alignment was performed by MEGA7.0.21 (Kumar et al., 2016) and visualized by GenDoc (Nicholas and Nicholas, 1997). Conserved percentage is shown as the following: black 100%, dark gray 80%. **(C)** Western blot of extracted proteins isolated at 6, 12, and ≥20 dap incubated with anit-PINa/b. Note the increase of the HIN protein abundance during barley endosperm development.

FIGURE S9 Negative control of microscopic studies using secondary antibody Alexa488^®^. Scale = 100 μm.

FIGURE S10 Quantification of the fluorescent signal of anti-PINa/PINb. **(A)** We used ImageJ to mark areas in aleurone (A), subaleurone (SA), and starchy endosperm (SE) at 12 and ≥20 dap for the mean intensity identification. **(B)** Bar blots of the fluorescent intensity of anti-PINa/b in all tissues at 12 and ≥20 dap and in A, SA, and SE at 12 and ≥20 dap. Scale is 100 μm. Areas (*n* = 8 for 12 dap; *n* = 7 for ≥20 dap) were quantified for A, SA, and SE from at least two slides with three sections. For statistical analyses, we performed a Student’s *t*-test. Bars represent standard deviation. Note the indicated *p*-values.

TABLE S1 Selected RGs are listed by their gene number, gene symbol, gene product function, gene ID (Unigene), primer sequence, amplicon length, corresponding mRNA sequence, corresponding protein (Uniprot) entry, and the identified protein in our MS data.

TABLE S2 Ranking of the RGs for spatio-temporal RT-qPCR analyses in the barley cultivar Golden Promise during caryopsis development.

TABLE S3 *HINa*, *HINb1*, and *HINb2* are listed by their gene symbol, gene product function, cDNA entry (Uniprot), primer sequence, amplicon length, corresponding protein (Uniprot) entry, and the identified protein in our MS data.

TABLE S4 MS data.
