# Supplementary figures and images for: Using RT-qPCR, Proteomics, and Microscopy to Unravel the Spatio-Temporal Expression and Subcellular Localization of Hordoindolines Across Development in Barley Endosperm
Source: Front Plant Sci. 2018 Jun 13;9:775. doi: 10.3389/fpls.2018.00775 (PMC6008550; doi:10.3389/fpls.2018.00775)

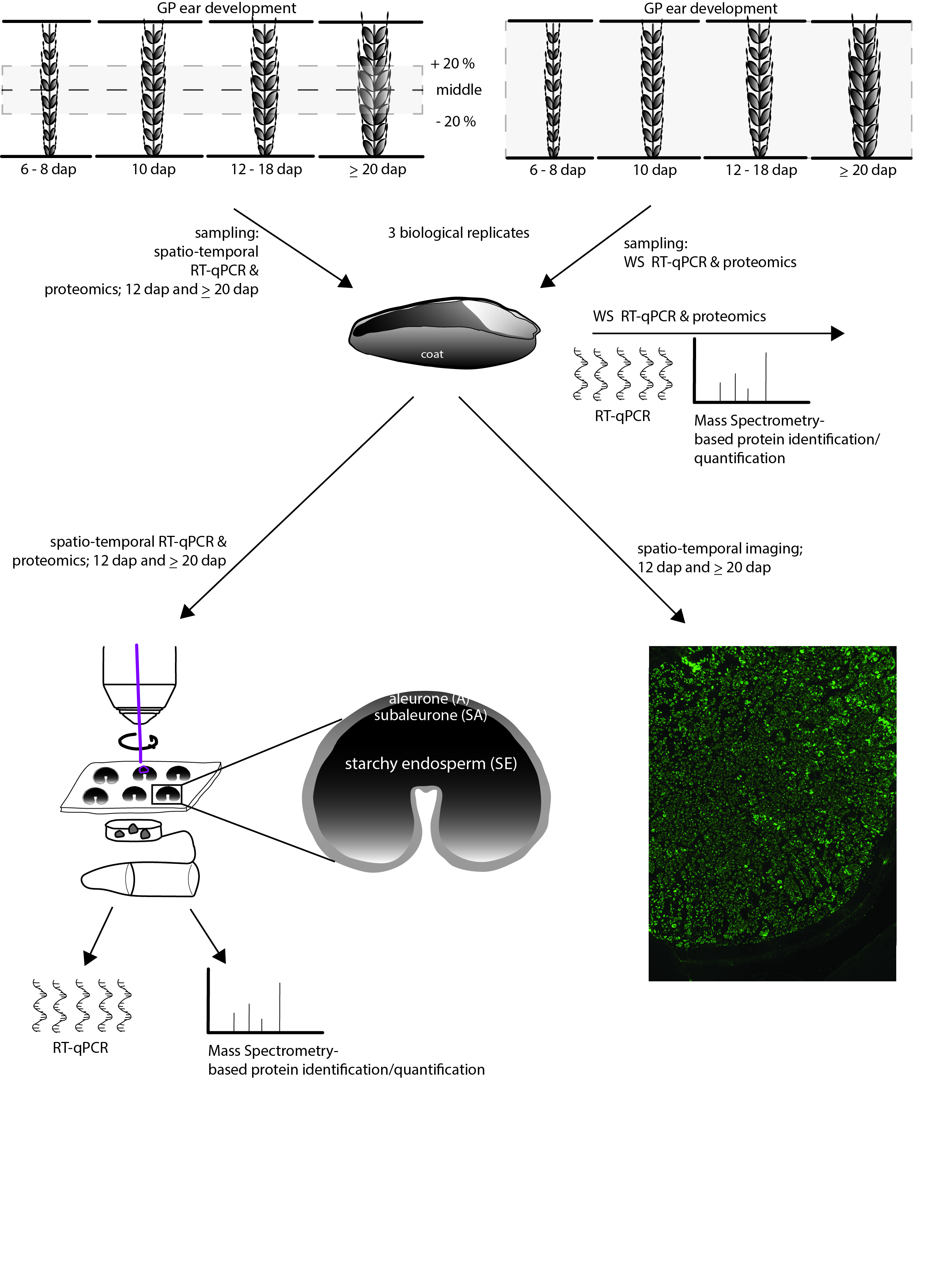

Supplement: FIGURE S1 — Schematic workflow of our multi-disciplinary approach as described in Section “Materials and Methods.” [file Image_1.TIF]

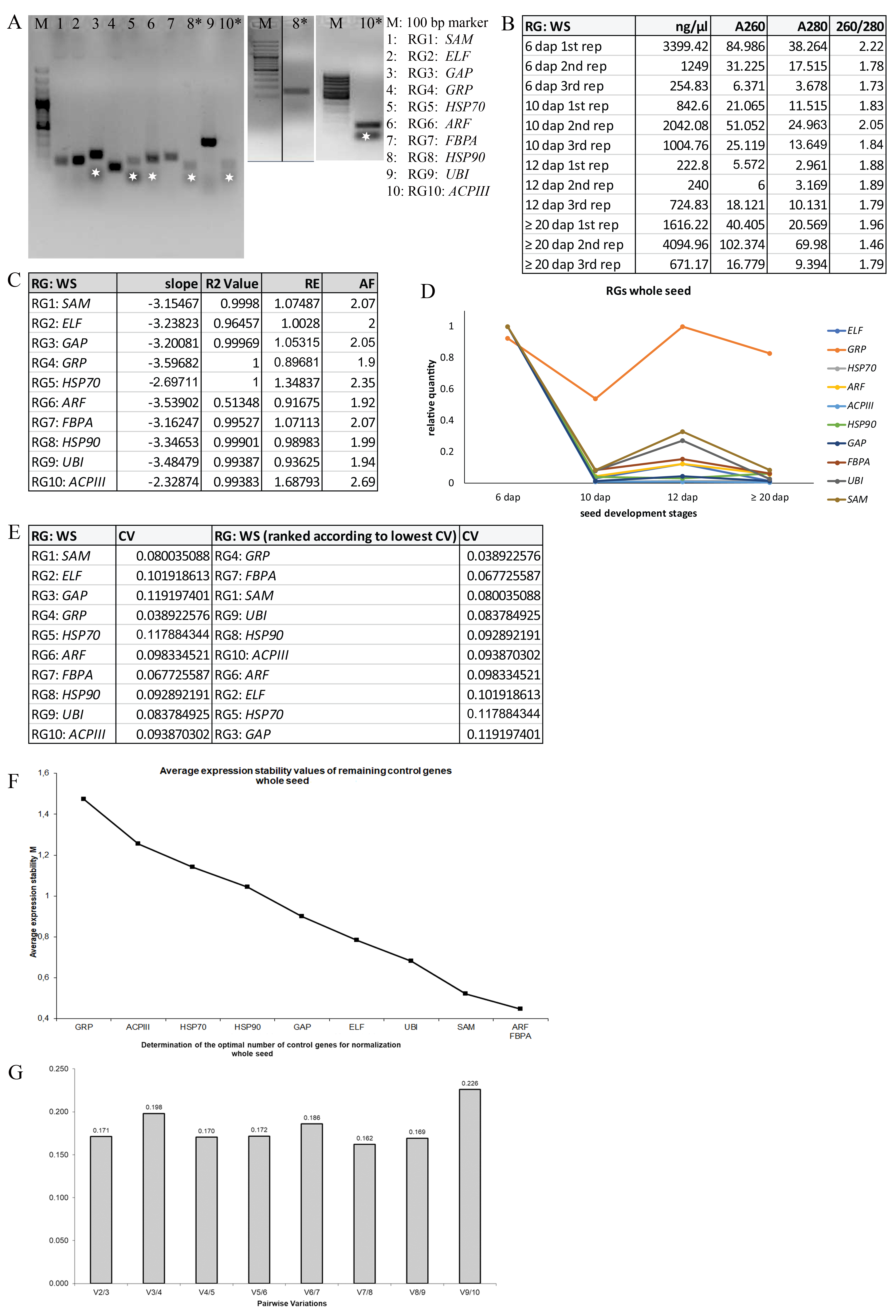

Supplement: FIGURE S2 — RT-qPCR reaction quality control and amplification efficiency of 10 candidate RGs for RT-qPCR in the barley cultivar GP during endosperm development. (A) Detection of cDNA of all RGs. M: DNA ladder 100 bp. Primer pairs are indicated by an asterisk. (B) Quantification of RNA extracted from seeds at 6, 10, 12, and ≥20 dap. RNA integrity was controlled photometrically (260/280). (C) Results from standard curves of the selected candidate RGs: slope, R2 value, reaction efficiency (RE), and amplification factor (AF). (D) Relative quantification of all RGs from the whole seed harvested at 6, 10, 12, and ≥20 dap. (E) Coefficient of variation (CV) of all RGs. (F) Average expression stability (M) of all RGs from the whole seed harvested at 6, 10, 12, and ≥20 dap calculated by geNorm. (G) Calculation of the pairwise variation by geNorm. [file Image_2.TIF]

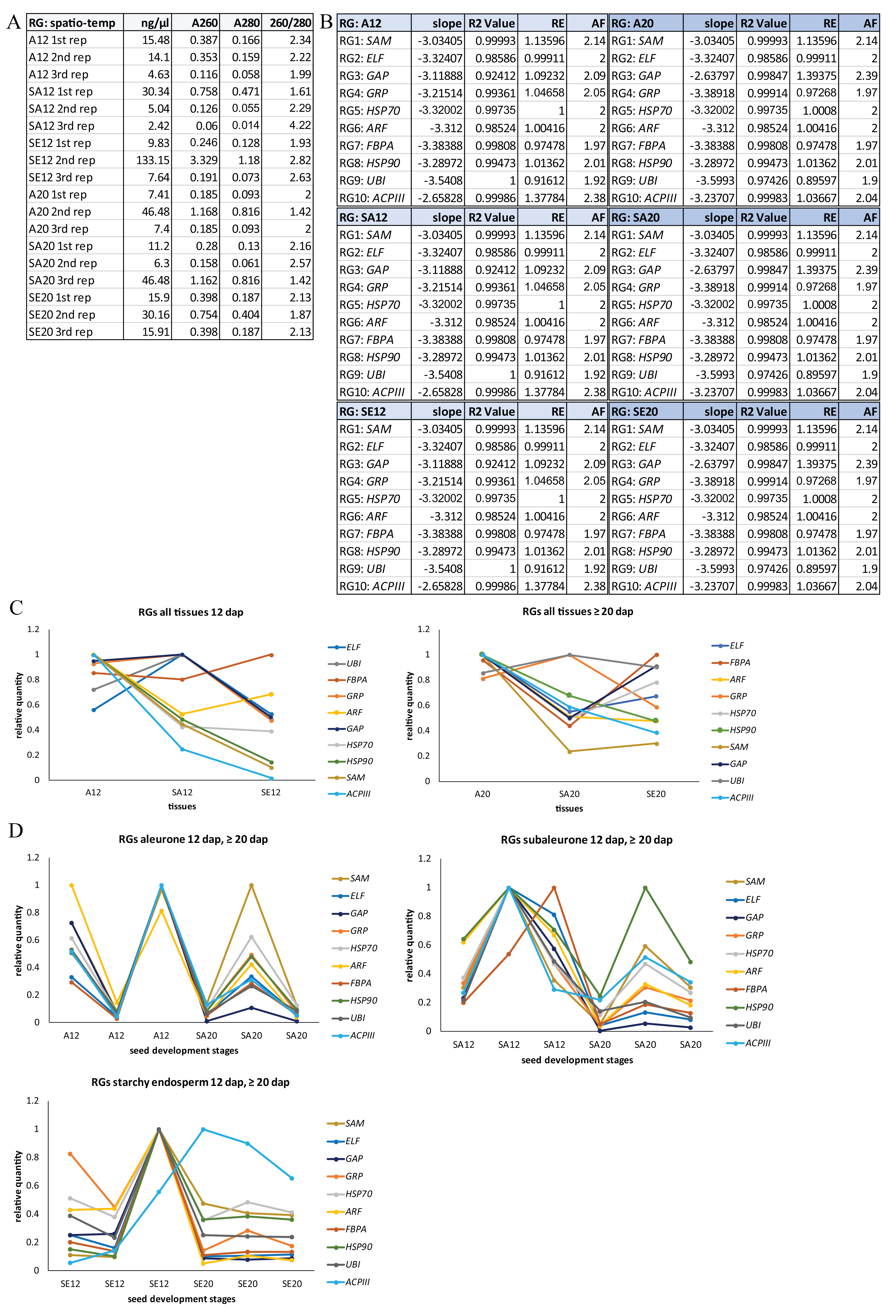

Supplement: FIGURE S3 — RT-qPCR reaction quality control and amplification efficiency of 10 candidate RGs for spatio-temporal RT-qPCR during GP endosperm development. (A) Quantification of RNA extracted from aleurone, subaleurone, and starchy endosperm at 12 and ≥20 dap. RNA integrity was controlled photometrically (260/280). (B) Results from standard curves of the selected candidate RGs: slope, R2 value, reaction efficiency (RE), and amplification factor (AF). (C) Relative quantification of all RGs from all tissues at 12 and ≥20 dap. (D) Relative quantification of all RGs from and from aleurone (A), subaleurone (SA), and starchy endosperm (SE) at 12 and ≥20 dap. [file Image_3.JPEG]

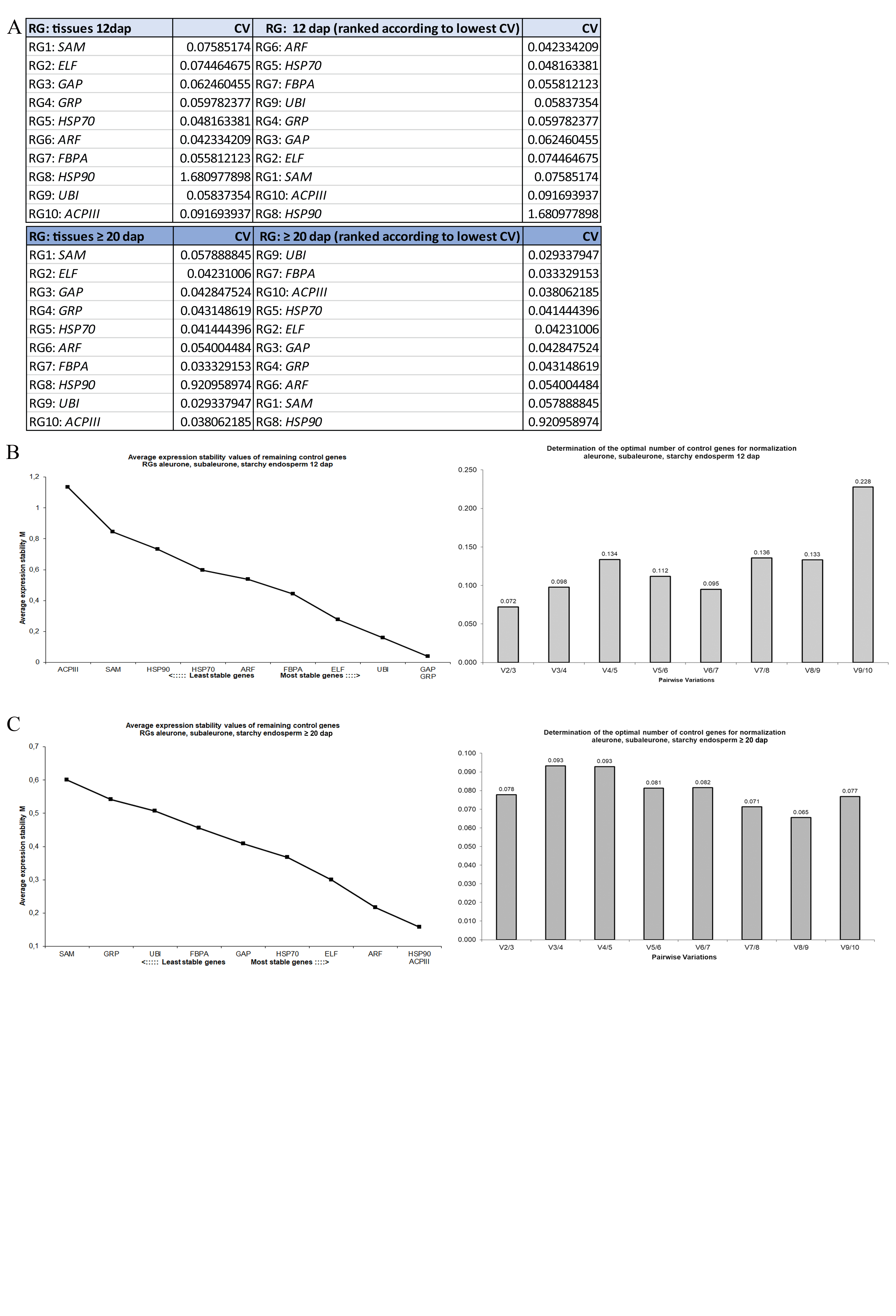

Supplement: FIGURE S4 — RT-qPCR reaction quality control and amplification efficiency of 10 candidate RGs for RT-qPCR in GP endosperm development at 12 and ≥20 dap for all tissues. (A) Coefficient of variation (CV) of all RGs for all tissues at 12 and ≥20 dap. (B) Average expression stability (M) of all RGs from the all tissues at 12 dap calculated by geNorm. Note the calculation of the pairwise variation by geNorm. (C) Average expression stability (M) of all RGs from the all tissues at ≥20 dap calculated by geNorm. Note the calculation of the pairwise variation by geNorm. [file Image_4.TIF]

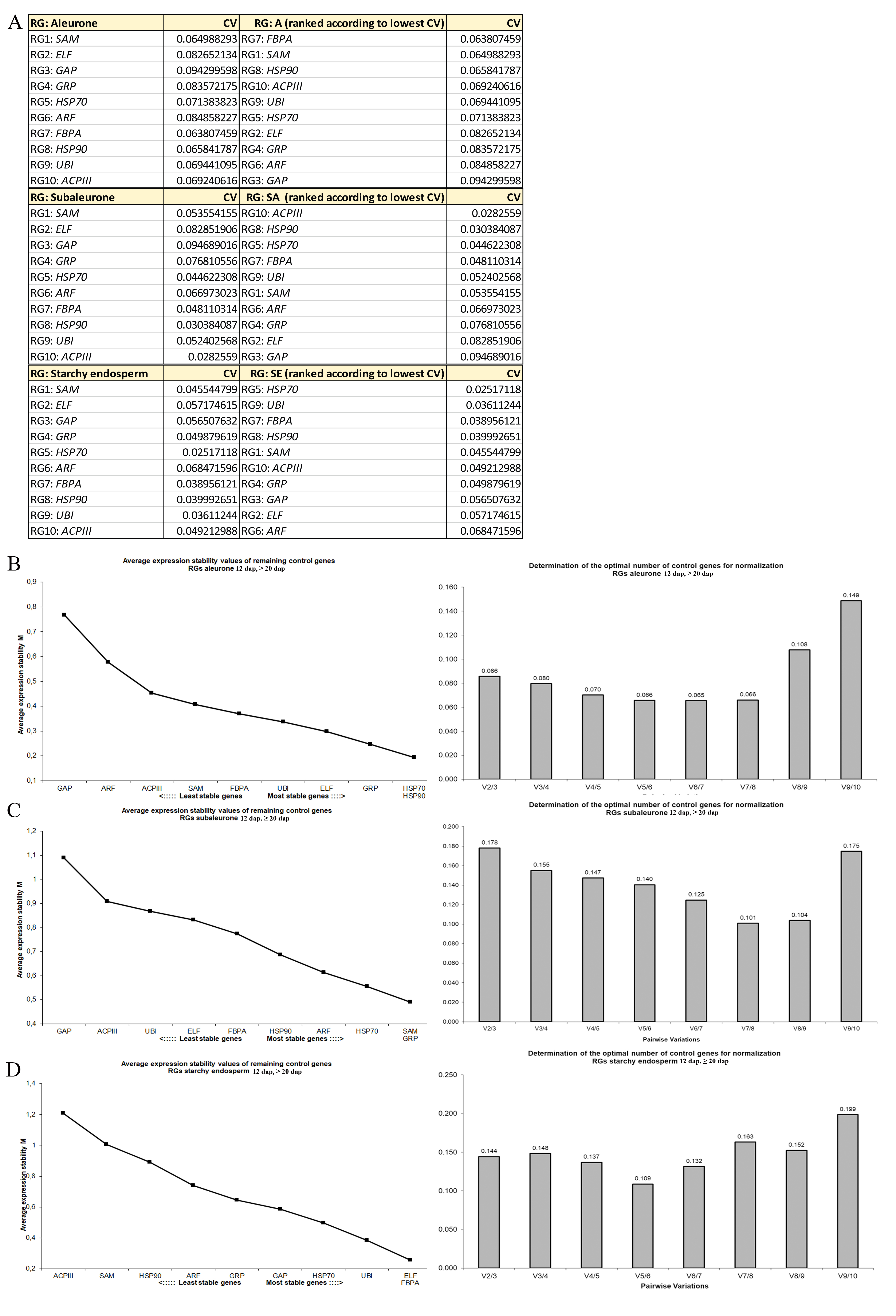

Supplement: FIGURE S5 — RT-qPCR reaction quality control and amplification efficiency of 10 candidate RGs for RT-qPCR in barley endosperm for aleurone (A), subaleurone (SA), and starchy endosperm (SE) at 12 and ≥20 dap. (A) Coefficient of variation (CV) of all RGs for A (12 dap, ≥ 20 dap), SA (12 and ≥20 dap), and SE (12 and ≥20 dap). Average expression stability (M) of all RGs for (B) A (12 and ≥20 dap), (C) SA (12 and ≥20 dap), and (D) SE (12 and ≥20 dap). Note the calculation of the pairwise variation by geNorm. [file Image_5.jpg]

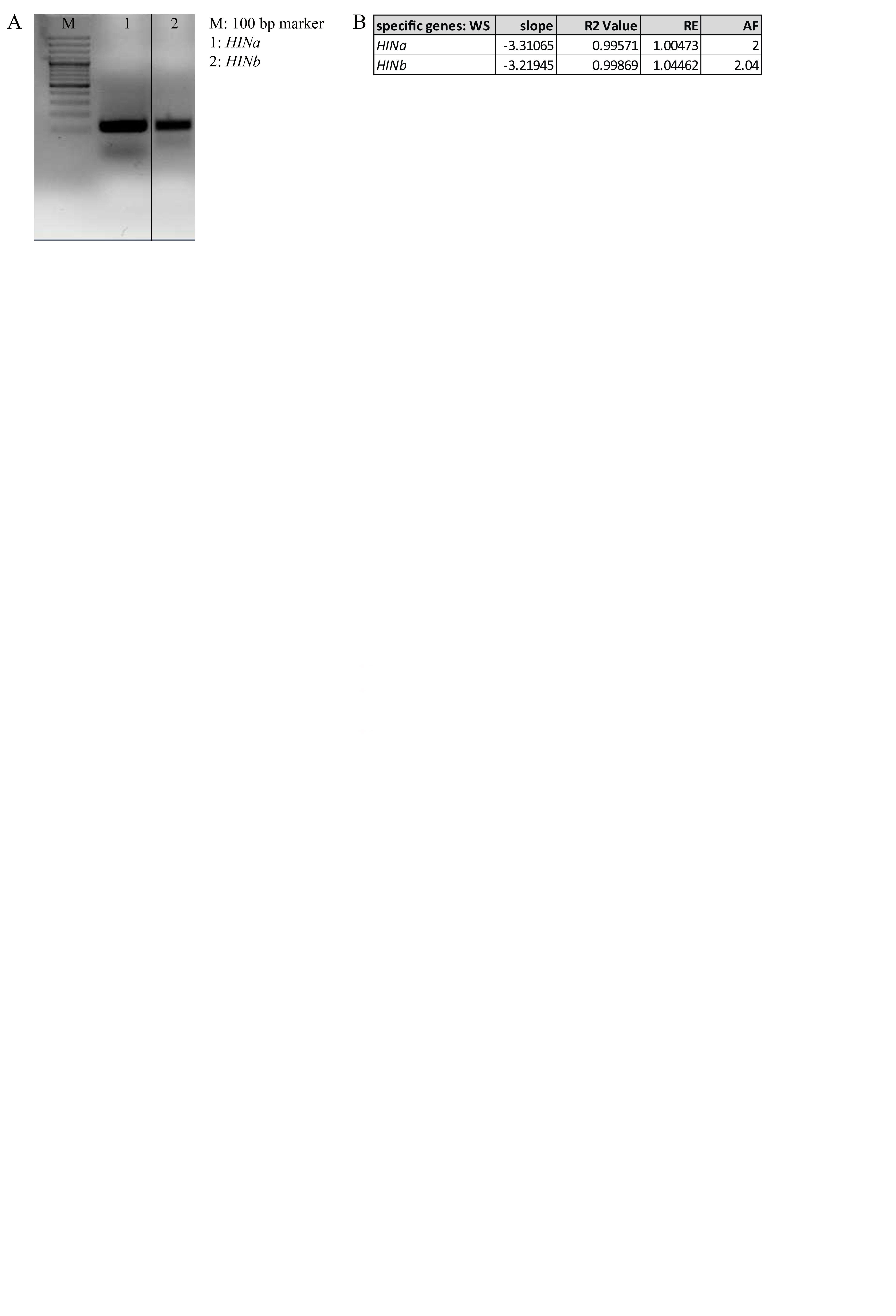

Supplement: FIGURE S6 — RT-qPCR reaction quality control and amplification efficiency of the HIN genes by RT-qPCR in barley endosperm. (A) cDNA amplification of HINa and HINb. M: DNA ladder 100 bp. (B) Results from standard curves of HINa and HINb: slope, R2 value, reaction efficiency (RE), and amplification factor (AF). [file Image_6.TIF]

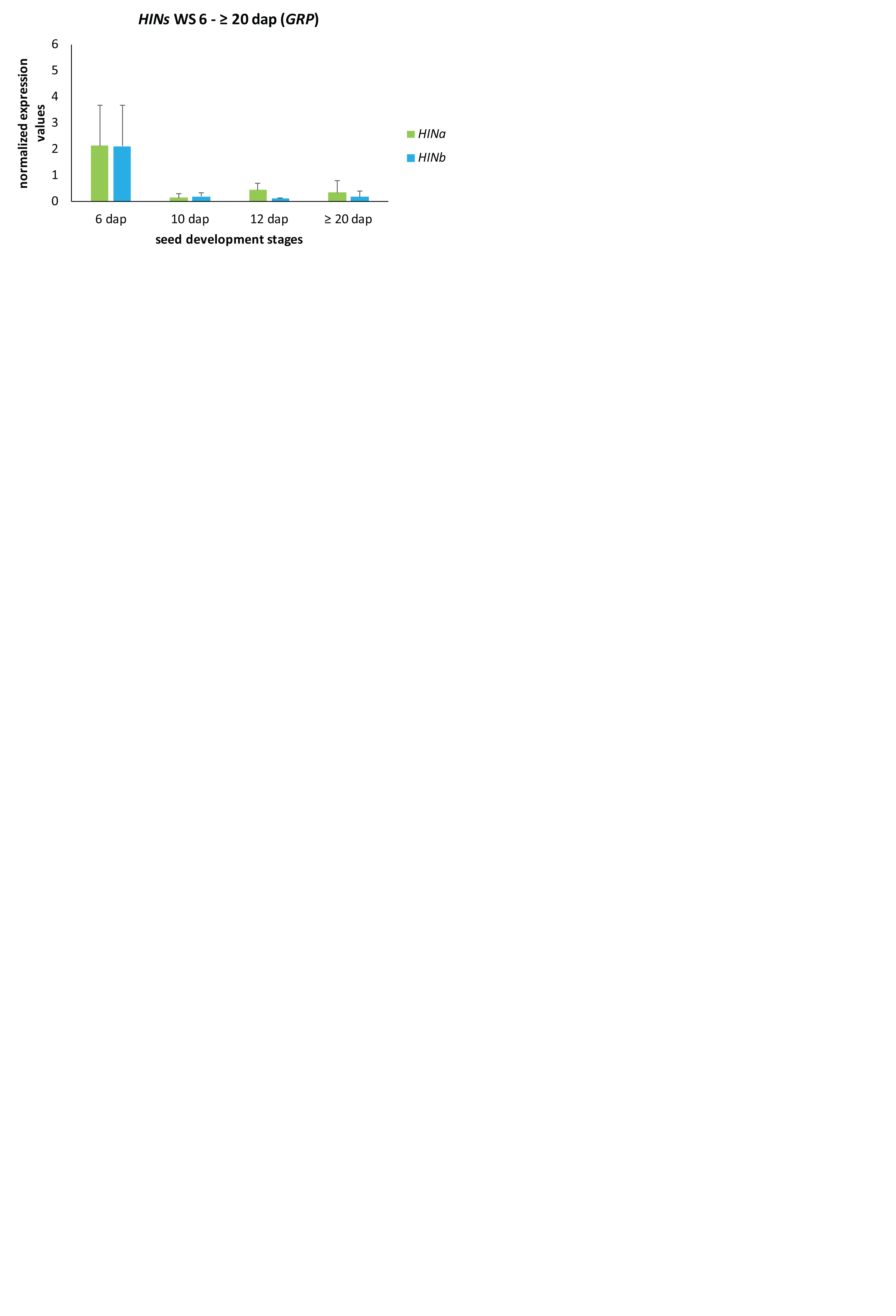

Supplement: FIGURE S7 — Bar graph describes the average over three biological replicates of the normalized transcripts from HINa and HINb at 6, 10, 12, and ≥20 dap with the most unstable RG (GRP). Bars represent standard deviation. [file Image_7.TIF]

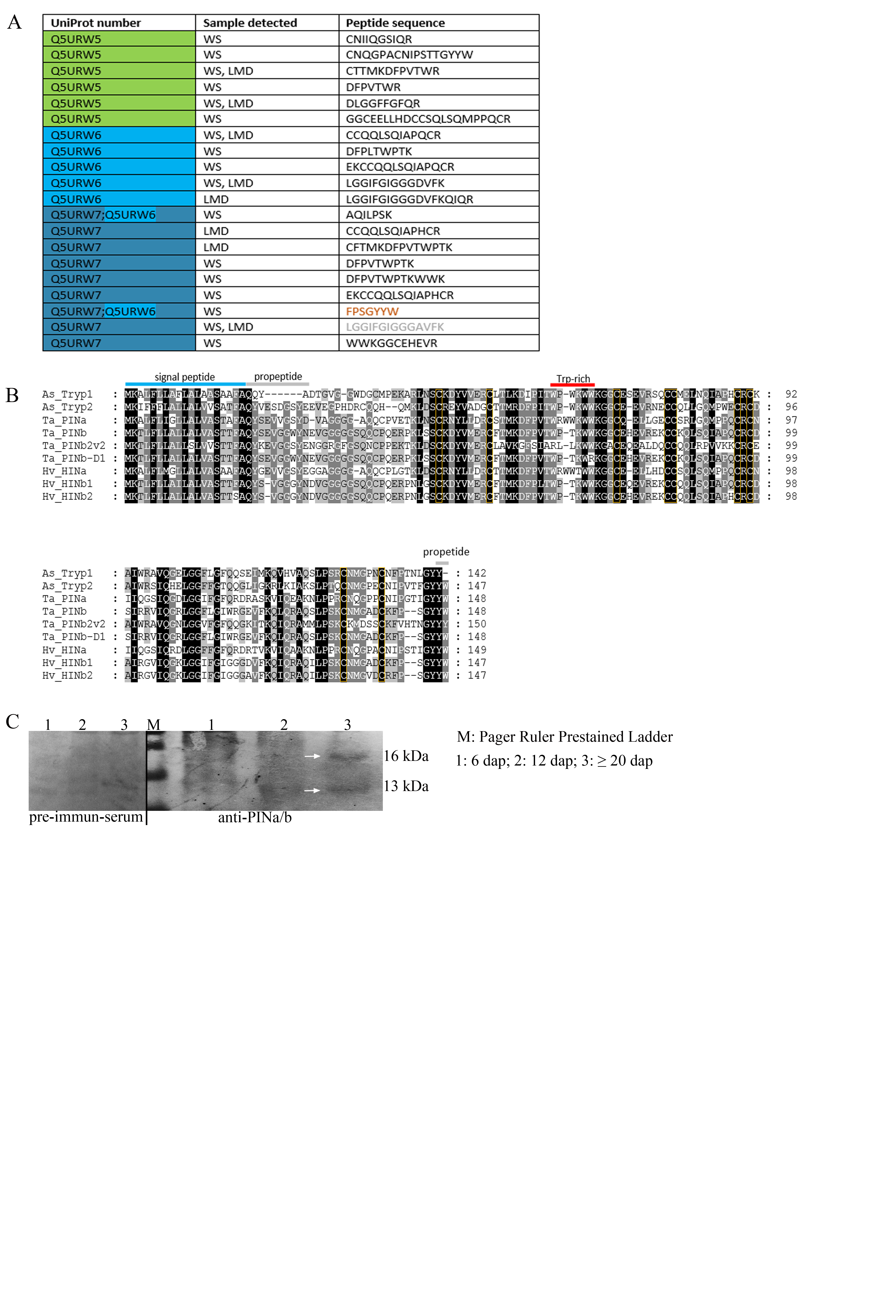

Supplement: FIGURE S8 — Identification and molecular characterization of HINa, HINb, and HINb2. (A) Identified peptides related to HINa (green), HINb1 (bright blue), and HINb2 (turquoise). (B) Protein alignment of the tryptophanins (oat), puroindolines (wheat), and hordoindolines (barley). Note the signal peptide, propeptide, the tryptophan-rich side, and the cysteine amino acids (orange box). Alignment was performed by MEGA7.0.21 (Kumar et al., 2016) and visualized by GenDoc (Nicholas and Nicholas, 1997). Conserved percentage is shown as the following: black 100%, dark gray 80%. (C) Western blot of extracted proteins isolated at 6, 12, and ≥20 dap incubated with anit-PINa/b. Note the increase of the HIN protein abundance during barley endosperm development. [file Image_8.TIF]

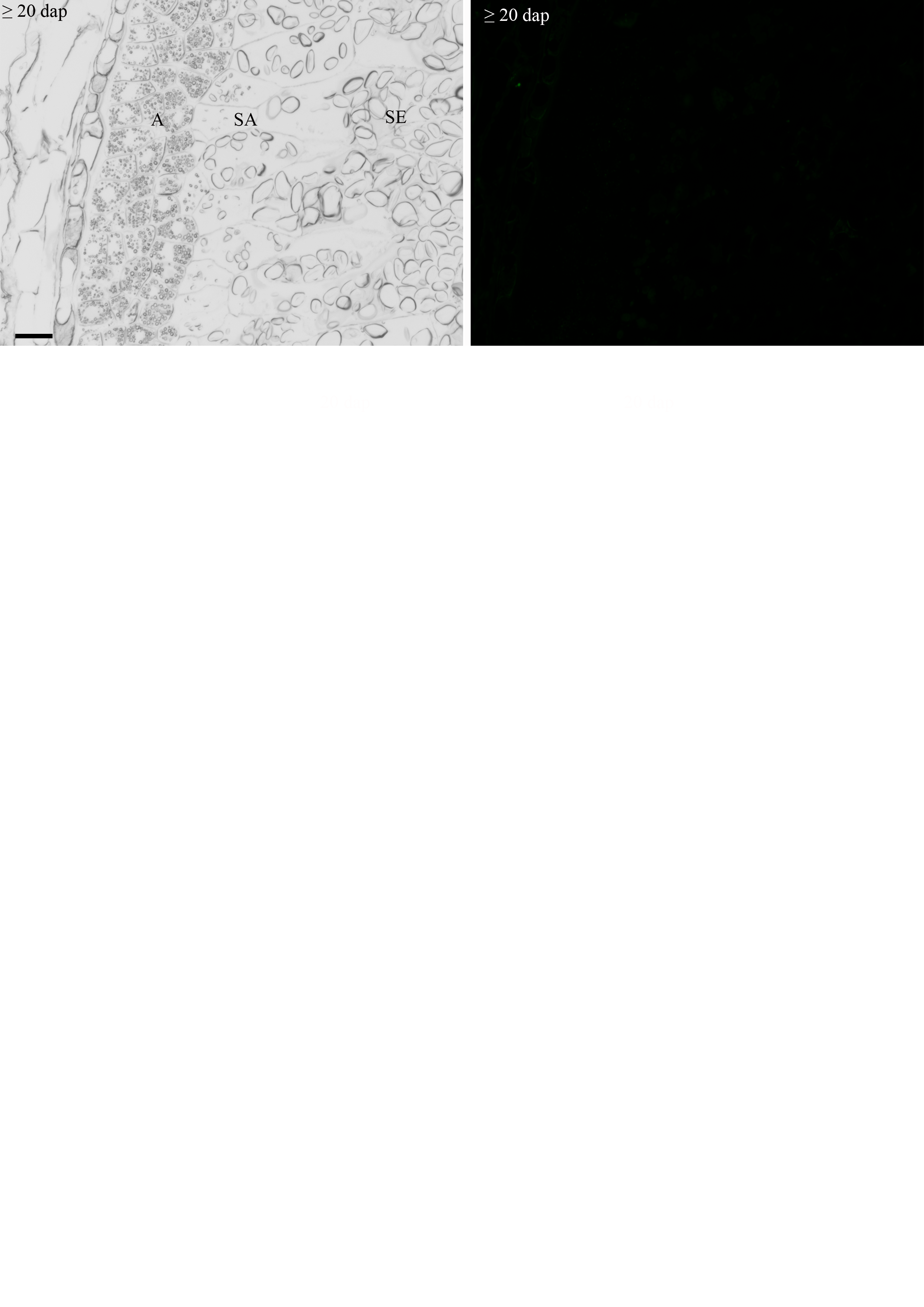

Supplement: FIGURE S9 — Negative control of microscopic studies using secondary antibody Alexa488®. Scale = 100 μm. [file Image_9.TIF]

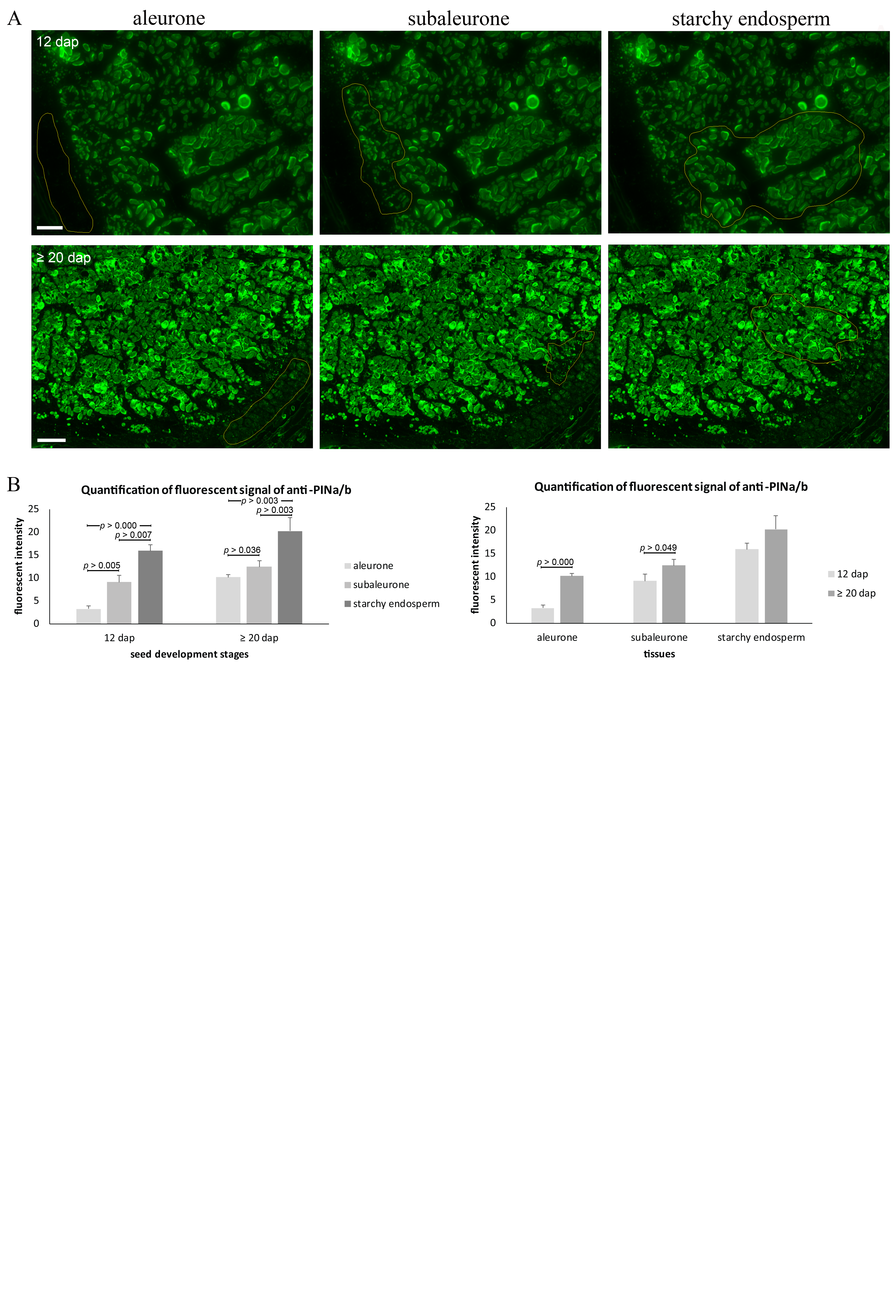

Supplement: FIGURE S10 — Quantification of the fluorescent signal of anti-PINa/PINb. (A) We used ImageJ to mark areas in aleurone (A), subaleurone (SA), and starchy endosperm (SE) at 12 and ≥20 dap for the mean intensity identification. (B) Bar blots of the fluorescent intensity of anti-PINa/b in all tissues at 12 and ≥20 dap and in A, SA, and SE at 12 and ≥20 dap. Scale is 100 μm. Areas (n = 8 for 12 dap; n = 7 for ≥20 dap) were quantified for A, SA, and SE from at least two slides with three sections. For statistical analyses, we performed a Student’s t-test. Bars represent standard deviation. Note the indicated p-values. [file Image_10.TIF]
